# A Synopsis of *Dicranum* Hedw. (Dicranaceae, Bryophyta) in China, with Special References to Four Species Newly Reported and Re-Evaluation of *Dicranum psathyrum* Klazenga

**DOI:** 10.3390/plants13131759

**Published:** 2024-06-25

**Authors:** Wen-Zhuan Huang, Chao Shen, Hao Xu, Lei Shu, Mamtimin Sulayman, Yu-Huan Wu, Rui-Liang Zhu

**Affiliations:** 1 College of Life and Environmental Sciences, Hangzhou Normal University, Hangzhou 311121, China; wzhmoss@163.com; 2 Bryology Laboratory, School of Life Sciences, East China Normal University, 500 Dongchuan Road, Shanghai 200241, China; shenchao_hznx@163.com (C.S.); 52261300061@stu.ecnu.edu.cn (H.X.); lshu@bio.ecnu.edu.cn (L.S.); 3 Laboratory of Biological Resources and Genetic Engineering, College of Life Science and Technology, Xinjiang University, Urumqi 830046, China; mamtimin@xju.edu.cn

**Keywords:** Bryophyta, Dicranales, new records, new synonym, phylogenetic analysis

## Abstract

*Dicranum* Hedw. is a highly diverse and widely distributed genus within Dicranaceae. The species diversity and distribution of this genus in China, however, remain not well known. A new revision of *Dicranum* in China using morphological and molecular phylogenetic methods confirms that China has 39 species, including four newly reported species, *D. bardunovii* Tubanova & Ignatova, *D. dispersum* Engelmark, *D. schljakovii* Ignatova & Tubanova, and *D. spadiceum* J.E.Zetterst. *Dicranum psathyrum* Klazenga is transferred to *Dicranoloma* (Renauld) Renauld as a new synonym of *Dicranoloma fragile* Broth. Two species, *Dicranum brevifolium* (Lindb.) Lindb. and *D. viride* (Sull. & Lesq.) Lindb. are excluded from the bryoflora of China. A key to the Chinese *Dicranum* species is also provided. These results indicate an underestimation of the distribution range of numerous *Dicranum* species, underscoring the need for further in-depth investigations into the worldwide *Dicranum* diversity.

## 1. Introduction

Dicranaceae are a family of mosses (Bryophyta). *Dicranum* Hedw. (including *Orthodicranum* (Bruch & Schimp.) Loeske) stands out as one of the most diverse genera within Dicranaceae, comprising approximately 98 accepted species [1]. The taxonomy of this genus presents significant challenges owing to the considerable variation of gametophytic characteristics influenced by environmental conditions [2,3,4,5]. Recent advancements in molecular phylogeny have led to a more precise delineation of *Dicranum*, including the identification of distinct groups such as *Dicranum* species with fragile leaves [6,7], *D. fuscescens* Turner complexes [8,9], *D. scoparium* Hedw. complexes [4,10], arctic *Dicranum* species [11], *D. acutifolium* (Lindb. & Arnell) C.E.O.Jensen complexes [12,13], and European *Dicranum* species [14]. Despite these valuable insights, it is essential to recognize the geographical limitations of these studies, which have predominantly focused on Europe and Asian Russia, thus restricting our comprehensive understanding of *Dicranum* species diversity in other regions worldwide. Particularly in China, the delineation and relationships among morphological species of *Dicranum* remain challenging to ascertain.

In 1890–1895, Italian missionary Giuseppe Giraldi collected numerous bryophyte specimens in Shaanxi Province, China. These specimens were studied by Carl Müller, and the findings were published in the work titled *Bryologia provinciae Schen-si Sinensis I-III* [15,16,17]. The discovery of *Dicranum rectifolium* Müll.Hal. and *D. drummondii* Müll.Hal. (reported as *D. thelinotum* Müll.Hal.) marked the first known presence of *Dicranum* in China in 1896 [15]. Subsequently, additional researchers such as Salmon [18], Paris [19], Brotherus [20,21,22], Reimers [23], and Bartram [24] documented various *Dicranum* species in China (Table 1). By the end of World War II, a total of sixteen *Dicranum* species had been recorded in China. Significantly, three of these, including *D. rectifolium* [15], *D. papillidens* Broth. [20], and *D. cheoi* E.B.Bartram [24], were newly described to science. Regrettably, no Chinese botanists had contributed to the early reports on *Dicranum* species in China.

After World War II, a research group led by Pan-Chieh Chen, a pioneer in Chinese modern bryology [25], embarked on exploring the Chinese bryoflora. The results of these investigations were published in various Chinese floras, offering detailed descriptions and illustrations of *Dicranum* species. The earliest regional moss flora study was completed in 1963 [26], documenting seven previously undocumented *Dicranum* species in China: *D. fulvum* Hook., *D. elongatum* Schleich. *ex* Schwägr., *D. groenlandicum* Brid., *D. fragilifolium* Lindb., *D. fuscescens* Turner, *D. undulatum* Schrad. *ex* Brid., and *D. spurium* Hedw. Subsequently, several species were reported new to China by other Chinese bryologists [27,28,29,30,31,32], resulting in a total of 43 recorded *Dicranum* species listed in the *Moss Flora of China* [33].

Notably, Gao et al. [34] raised *Dicranum* subgenus *Pseudochorisodontium* Broth. as a new genus, *Pseudochorisodontium* (Broth.) C.Gao, and recent studies [3,7,14,35,36,37,38] have embraced a broader concept of this genus, merging *Orthodicranum* into *Dicranum*. Following these treatments, 34 *Dicranum* species have been cataloged in the *Species Catalogue of China* [35,36]. Nevertheless, there are still numerous omissions in this checklist that necessitate further study and updating. For example, more recently, Huang et al. [7,38] described two new species and a newly recorded species in China: *D. baicalense* Tubanova, *D. hengduanense* W.Z.Huang & R.L.Zhu, and *D. shennongjiaense* W.Z.Huang & R.L.Zhu (Table 1).

**Table 1:** A list in chronological order of the *Dicranum* species described and recorded from China. Names in bold indicate being newly reported to science based on the specimens collected from China. *Dicranum psathyrum* (≡*Dicranoloma fragile* Klazenga) is not included.

No.	Species	Year	Reference and Notes
1	***Dicranum rectifolium***	1896	Müller [15]
2	*Dicranum drummondii*	1896	Müller [16], reported as *Dicranum thelinotum*
3	*Dicranum japonicum*	1897	Müller [17], reported as *Dicranum schensianum*
4	*Dicranum montanum*	1898	Müller [17]
5	*Dicranum hamulosum*	1900	Salmon [18], reported as *Dicranum crispofalcatum*
6	*Dicranum lorifolium*	1900	Salmon [18]
7	*Dicranum nipponense*	1908	Paris [19], reported as *Dicranum rufescens*
8	***Dicranum papillidens***	1924	Brotherus [20]
9	*Dicranum scoparium*	1929	Brotherus [21], reported as *Dicranum orthophyllum*
10	*Dicranum muehlenbeckii*	1929	Brotherus [21]
11	*Dicranum crispifolium*	1929	Brotherus [21]
12	*Dicranum kashmirense*	1929	Brotherus [21]
13	*Dicranum majus*	1929	Brotherus [21]
14	*Dicranum mayrii*	1929	Brotherus [22], reported as *Dicranum formosicum*
15	*Dicranum flagellare*	1931	Reimers [23]
16	***Dicranum cheoi***	1935	Bartram [24]
17	*Dicranum fulvum*	1963	Chen [26]
18	*Dicranum elongatum*	1963	Chen [26]
19	*Dicranum groenlandicum*	1963	Chen [26]
20	*Dicranum fragilifolium*	1963	Chen [26]
21	*Dicranum fuscescens*	1963	Chen [26]
22	*Dicranum undulatum*	1963	Chen [26]
23	*Dicranum spurium*	1963	Chen [26]
24	*Dicranum bonjeanii*	1963	Li [27]
25	*Dicranum polysetum*	1977	Gao [28]
26	*Dicranum scottianum*	1977	Gao [28]
27	*Dicranum linzianum*	1979	Gao and Chang [29]
28	*Dicranum himalayanum*	1985	Li [30]
29	*Dicranum assimicum*	1985	Li [30]
30	*Dicranum setifolium*	1992	Gao and Cao [31]
31	*Dicranum leiodontum*	1992	Gao and Cao [31]
32	*Dicranum hakkodense*	2008	Ignatova and Fedosov [6]
33	***Dicranum hengduanense***	2023	Huang et al. [7]
34	***Dicranum shennongjiaense***	2024	Huang et al. [38]
35	*Dicranm baicalense*	2024	Huang et al. [38]
36	*Dicranum bardunovii*	—	Present study
37	*Dicranum dispersum*	—	Present study
38	*Dicrnaum schljakovii*	—	Present study
39	*Dicranum spadiceum*	—	Present study

To advance our understanding of *Dicranum* diversity in China, we conducted a taxonomic revision of *Dicranum* in China over the last two years [7,38]. Throughout this period, we encountered some intriguing specimens that did not correspond to any previously documented *Dicranum* species in China. To clarify the taxonomic classification of these Chinese mosses, we conducted a phylogenetic analysis by sequencing five chloroplast markers (*rpo*B, *rps*4-*trn*T, *rps*19-*rpl*2, *trn*H-*psb*A, and *trn*L-*trn*F) and one nuclear marker (ITS region). Through a meticulous examination of morphological characteristics and molecular phylogeny analysis, we confirmed the existence of four new records of *Dicranum* species in China, including *D. bardunovii*, *D. dispersum*, *D. schljakovii*, and *D. spadiceum.*

The primary objectives of this study are (1) to confirm the species diversity of *Dicranum* in China based on literature sources and our findings, (2) to provide photographs and descriptions of four new national records of *Dicranum* species, (3) to delineate the distribution of each *Dicranum* species within China and elucidate the differences between each species and those commonly confused species, and (4) to provide an updated key to *Dicranum* species in China.

## 2. Results

### 2.1. Results of Phylogenetic Analysis

**Figure 1:**
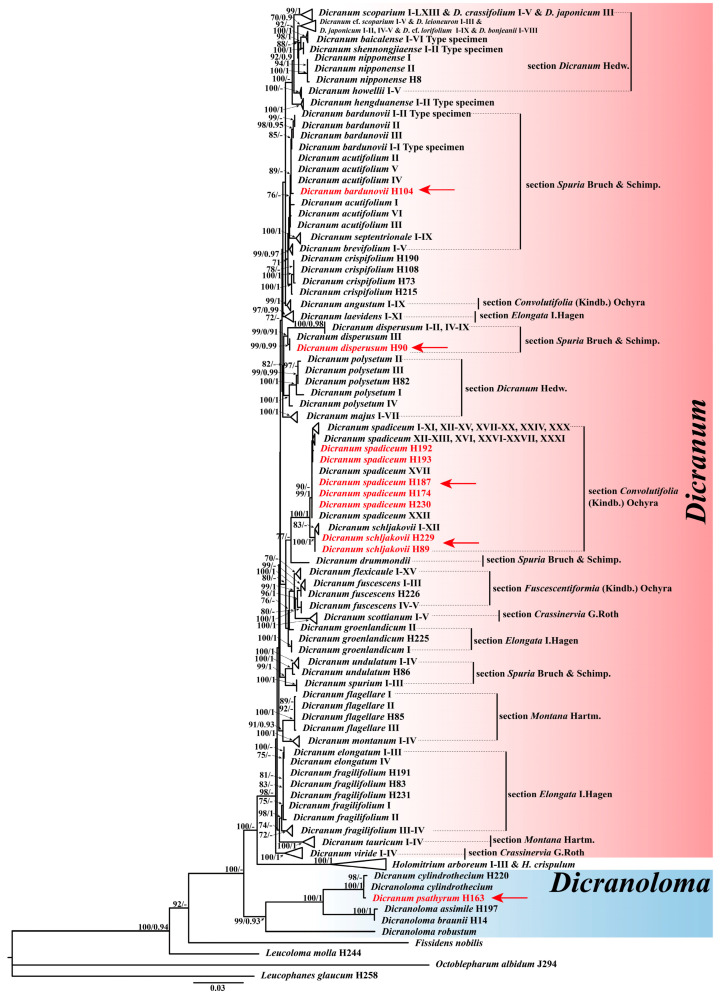
Phylogeny of *Dicranum* species inferred from the combined dataset (*trn*H*-psb*A, *rps*4*-trn*T, *trn*L*-trn*F, *rps*19*-rpl*2, *rpo*B, and ITS). The topology derived from the best*-*scoring ML tree in IQtree is shown. ML bootstrap values BS ≥ 70 are shown on the left and Bayesian posterior probabilities values PP ≥ 0.90 on the right. The subject species are in red and indicated by arrows. Section division refers to Hodgetts et al. [37].

The aligned six-loci dataset comprised 2692 characters, consisting of the following segments and lengths: nrITS1-5.8S-ITS2 (784 bp), *rps*19-*rpl*2 (352 bp), *rpo*B (457 bp), *rps*4-*trn*T (510 bp), *trn*H-*psb*A (139 bp), and *trn*L-*trn*F (450 bp). Of the 2692 aligned nucleotides in the 309 accessions analyzed, 1662 were constant sites, 400 were singleton sites, and 630 were parsimony-informative. Maximum likelihood (ML) and Bayesian inference (BI) analyses produced congruent trees with robust support for the majority of nodes. The ML tree topology with bootstrap values (BS_ML_ and PP_BI_) is shown in Figure 1.

The phylogeny positions of *Dicranum* samples collected in China are as follows (Figure 1): (1) *D. bardunovii*, *D. angustum*, *D. acutifolium*, *D. brevifolium*, *D. septentrionale*, and *D. crispifolium* constitute a weakly supported clade. Within this clade, nine accessions of *D. angustum* (BS_ML_ = 99, PP_BI_ = 1), five accessions of *D. brevifolium* (BS_ML_ = 99, PP_BI_ = 0.97), four accessions of *D. crispifolium* (BS_ML_ = 100, PP_BI_ = 1), and nine accessions of *D. septentrionale* (BS_ML_ = 99, PP_BI_ = 0.97) form highly supported subclades, respectively. However, the accession of *D. bardunovii* from China is nested deeply within *D. acutifolium*. (2) Ten accessions of *D. dispersum* form a strongly supported clade (BS_ML_ = 99, PP_BI_ = 0.91). Within this clade, one accession from China and one accession from Russia form a single subclade with high support values (BS_ML_ = 99, PP_BI_ = 0.99). (3) Two accessions of *D. schljakovii* collected from China form a well-supported clade with twelve other accessions (BS_ML_ = 100, PP_BI_ = 1). (4) Five accessions of *D. spadiceum* collected from China (Qinghai and Xinjiang) are nested deeply within the other accessions from Europe and Russia (BS_ML_ = 100, PP_BI_ = 0.99). (5) The accession of *D. psathyrum* is deeply nested within the genus *Dicranoloma* (Renauld) Renauld (BS_ML_ = 99, PP_BI_ = 0.93).

Based on the phylogenetic analysis, we conclusively confirm the newly recorded distribution of *Dicranum dispersum*, *D. schljakovii*, and *D. spadiceum* in China, and propose transferring *D. psathyrum* to the genus *Dicranoloma*. In addition, the tree topology allows for considering the accession of *Dicranum bardunovii* from China within *D. acutifolium* (Figure 1). Despite this, significant morphological distinctions exist between these two species (see note under *D. bardunovii* for details), supporting that our collection in China represents *D. bardunovii* rather than *D. acutifolium*.

### 2.2. Newly Recorded Species for China

**Figure 2:**
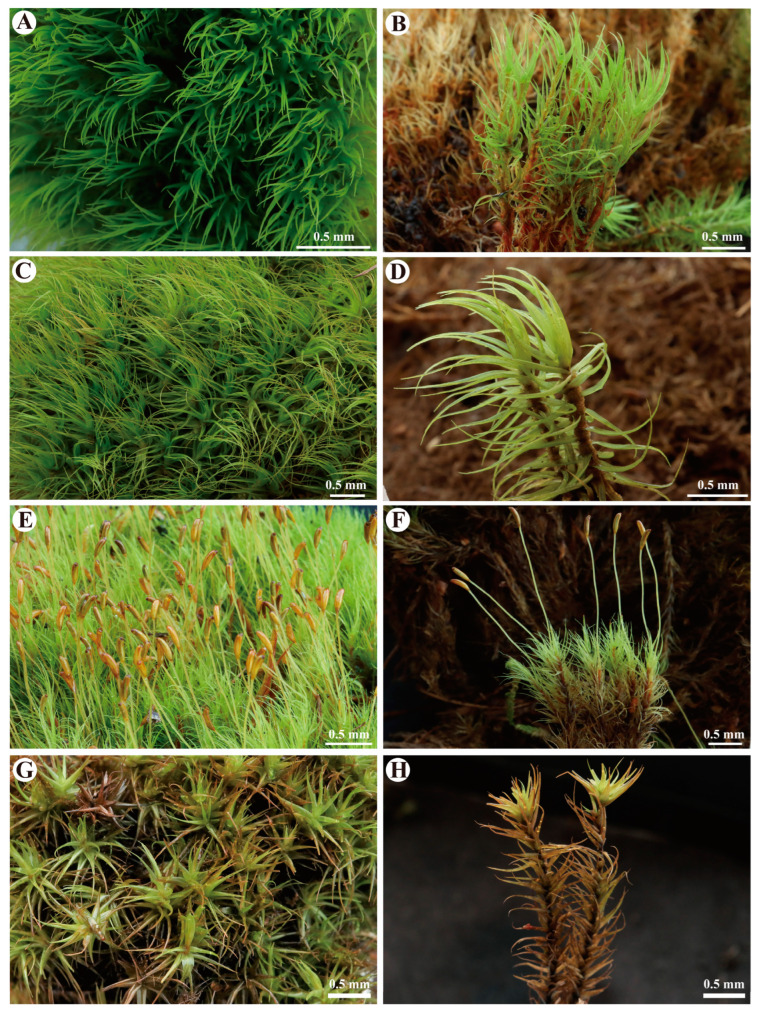
Plants of *Dicranum* species new to China. (**A**,**B**) *D. bardunovii* Tubanova & Ignatova, from *R.L. Zhu* et al. *20220830-30B* (HSNU); (**C**,**D**) *D. dispersum* Engelmark, from *S.B. Zhang 20220709-41* (HSNU); (**E**,**F**) *D. schljakovii* Ignatova & Tubanova, from *R.L. Zhu* et al. *20220803-314A* (HSNU); (**G**,**H**) *D. spadiceum* J.E. Zetterst., from *L. Shu & W.Z. Huang 20220817-38* (HSNU).

**Figure 3:**
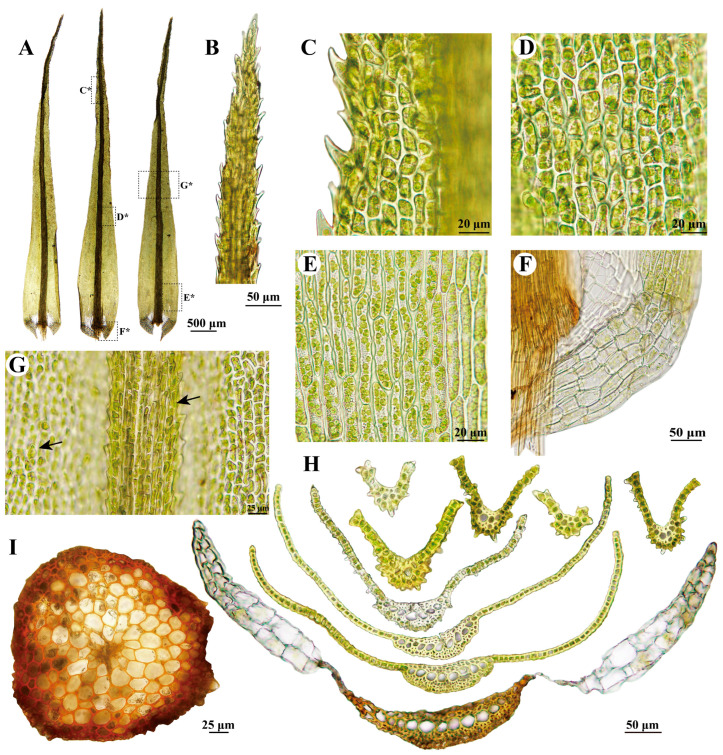
***Dicranum bardunovii*** Tubanova & Ignatova. All from *R.L. Zhu* et al. *20220830-30B* (HSNU). (**A**) Leaves, C*, D*, E*, F* indicates the position of C, D, E, F on the leaf, respectively; (**B**) Leaf apex; (**C**) Upper laminal cells of leaf; (**D**) Middle laminal cells of leaf; (**E**) Basal lamianl cells of leaf; (**F**) Alar cells of leaf; (**G**) Costa surface and laminal cells in the upper portion of leaf (in dorsal view), arrows shows papillose; (**H**) cross-sections of leaf; (**I**) cross-section of stem.

***Dicranum bardunovii*** Tubanova & Ignatova, Arctoa 20: 185. 2011 (Figure 2A,B and Figure 3).

**Distribution in China:** Yunnan (present report), new to China.

**Specimens examined: China. Yunnan.** Diqingzangzu Autonomous Prefecture, Deqin country, Baimaxueshan National Nature Reserve, at Baimaxueshan Pass, 28°22′52.68″ N, 99°1′12.03″ E, 4365 m, on soil near the base of azalea, 30 August 2022, *R.L. Zhu* et al. *20220830-30B* (HSNU).

**Description:** Plants robust, in loose tufts, green or yellow-green in the upper part, brownish below. Stems 5–8 cm, matted with white tomentum in the upper part, rusty tomentum and rhizoids at the base. Cross-section of stem is rounded, consisting of 1–2 layers of smaller, obviously thick-walled outer cortical cells, numerous rows of larger and thinner-walled inner cortical cells, and 6–9 rows of central strand cells. Leaves straight and erect-spreading when wet, slightly curved when dry, 5.8–7.5 × 0.75–0.86 mm, lanceolate, from ovate base gradually acuminate. Leaf margins are serrated in the upper 3/5, strongly serrated in the distal part, and entirely smooth below. Costa ca. 200 µm wide at the leaf base, about 1/5 of leaf width at the base, the dorsal side of the costa is sharply and densely scabrose in the upper part, with one row of guide cells in the transverse section, two stereid bands, ventral epidermis not differentiated, and well differentiated dorsal epidermis. Leaf lamina is unistratose, with bistratose regions on or near the upper margins. Upper and median laminal cells are 7–18 × 7–9 µm, quadrate, irregularly angled, or short rectangular, a lot of which are strongly prorulose on the upper ends on the dorsal side of the leaf. Basal laminal cells are elongated rectangular to linear, 43–100 × 7–10 µm, and pitted. Alar cells are differentiated, brownish, decurrent, and not extending to the costa, with a group of thin-walled hyaline cells between the alar cells and costa, with 2–3 layers of cells in the transverse section. Sporophyte not seen.

**Note:** This species was previously known only from Russia (including Asiatic Russia east of Yenissey River and the European part of the Urals) [9,37]. In China, it grows on stone at high altitudes up to 4365 m, usually associated with *Brachythecium coarctum* (Müll.Hal.) Ignatov & Huttunen, *Paraleucobryum enerve* (Thed.) Loeske, and *Symblepharis vaginata* (Hook. *ex* Harv.) Wijk & Margad.

*Dicranum bardunovii* is highly similar to *D. acutifolium*, but can be differentiated by its sharply and densely scabrose laminal cells and costa in the distal part of the leaf, whereas the latter species has smooth cells and costa [9]. Although Tubanova and Ignatova [9], Lang et al. [12], and Kiebacher and Szövényi [13] provided molecular evidence supporting distinctions between the two taxa, the phylogeny trees in their results exhibited weak clade support. Lang et al. [12] also found that one of the two accessions from the holotype specimen of *D. bardunovii* nested deeply within the clade of *D. acutifolium* and attributed it to mixed collections of these two species in the holotype specimen. However, the morphological characteristics of the type of specimen do not support their hypothesis (refer to Supplementary Table S2 in Lang et al. [12]). The phylogenetic tree in our analysis showed low branch support in this complex, with *D. bardunovii* collected from China deeply nested within *D. acutifolium* (Figure 1). However, morphologically, we are highly confident that the Chinese specimen belongs to *D. bardunovii*, as evidenced by the sharply and densely scabrose laminal cells and costa in the distal part of the leaf (Figure 3G).

**Figure 4:**
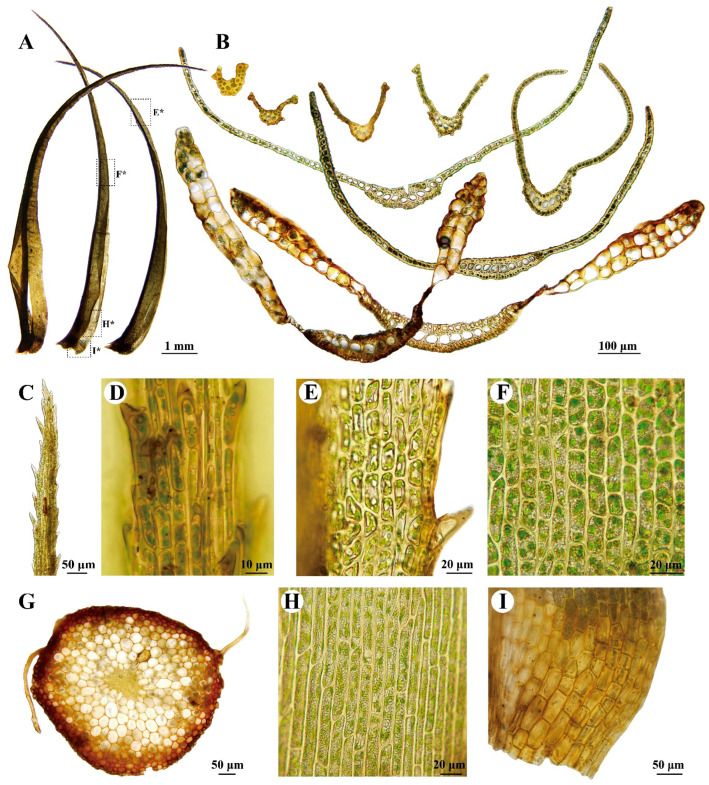
***Dicranum dispersum*** Engelmark. All from *S.B. Zhang 20220709-41* (HSNU). (**A**) Leaves, E*, F*, H*, I* indicates the position of E, F, H, I on the leaf, respectively; (**B**) Cross-sections of leaf; (**C**) Leaf apex; (**D**) Costa in the upper portion of leaf (dorsal view); (**E**) Upper laminal cells of leaf; (**F**) Middle laminal cells of leaf; (**G**) Cross-section of stem; (**H**) Basal laminal cells of leaf; (**I**) Alar cells of leaf.

**Figure 5:**
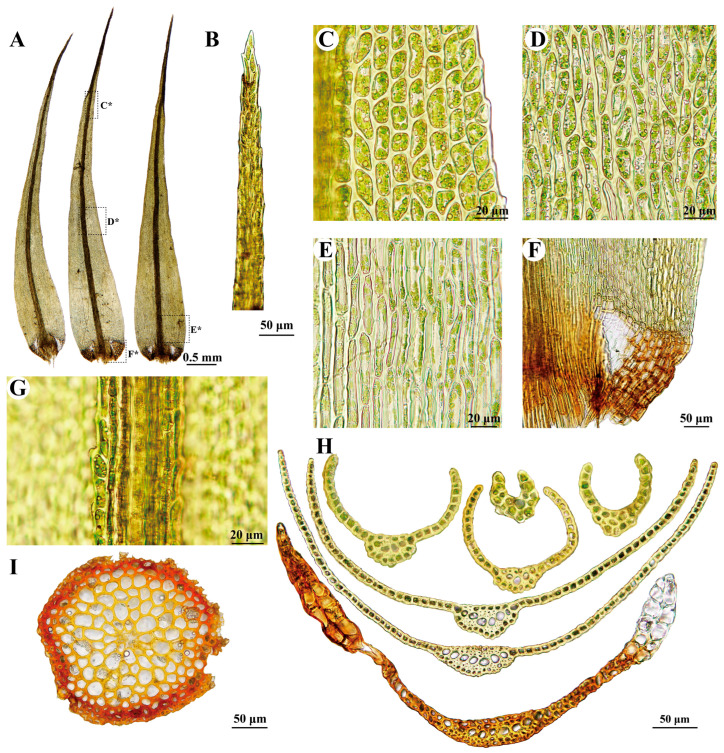
***Dicranum schljakovii*** Ignatova & Tubanova. All from *R.L. Zhu* et al. *20220803-314A* (HSNU). (**A**) Leaves, C*, D*, E*, F* indicates the position of C, D, E, F on the leaf, respectively; (**B**) Leaf apex; (**C**) Upper lamianl cells of leaf; (**D**) Middle lamianl cells of leaf; (**E**) Basal lamianl cells of leaf; (**F**) Alar cells of leaf; (**G**) Costa in upper portion of leaf (dorsal view); (**H**) Cross-sections of leaf; (**I**) Cross-section of stem.

*Dicranum bardunovii* is well characterized by (1) its ventral stereid band being exposed and surface cells not differentiated (Figure 3H), (2) a transverse section of leaf in the upper part like a pair of tongs (Figure 3H), (3) a sharply and densely scabrose dorsal side of laminal cells and costa in the distal part of the leaf (Figure 3G), (4) strongly serrated margins in the distal part (Figure 3C), and (5) bistratose alar cells (Figure 3H). There are no obvious differences between the morphological characteristics mentioned above from China and the illustrations based on the type of specimen in Tubanova and Ignatova [9].

***Dicranum dispersum*** Engelmark, Stuttgarter Beitr. Naturk., A 592: 4. 1999. (Figure 2C,D and Figure 4)

**Distribution in China:** Qinghai (present report), new to China.

**Specimens examined: China. Qinghai.** Tongren City. Zhamao Country, Maixiu area of Sanjiangyuan National Nature Reserve, 35°22′2″ N, 101°50′8″ E, 2942 m, southwest-facing slope 50°, on stone in a valley, 9 July 2022. *S.B. Zhang 20220709-41* (HSNU).

**Description:** Plants rather robust, in loose tufts, green in the upper part, and brownish below. Stems 4–8 cm, matted with white tomentum in the upper part, and rusty tomentum and rhizoids at the base. Cross-section of stem is rounded, consisting of 2–4 layers of smaller, obviously thick-walled outer cortical cells, numerous rows of larger and thinner-walled inner cortical cells, and a number of central strand cells. Leaves somewhat flexuous, erect-spreading when wet, loosely curved when dry, usually keeled in the upper part, and appearing V-shaped or looking like a pair of tongs in the cross-section. Leaves gradually narrowed from an ovate–lanceolate base to a long acuminate apex, 9.5–12 × 1–1.2 mm. Leaf margins are serrated in the upper 3/5 to 1/2, and entirely smooth below. Costa is 1/5–1/4 of leaf width at the base and 200–300 µm wide at the leaf base, with teeth on the dorsal surface in the upper part, with one row of guide cells in the transverse section, two stereid bands, and differentiated dorsal and ventral layers of cells. Leaf laminal is unistratose, with bistratose regions on or near the upper margins. Upper and median laminal cells are 13–25(–34) × 9–11 µm, quadrate, irregularly angled, short rectangular, smooth, or slightly mammillose. Basal laminal cells are elongated–rectangular to linear, 65–120 × 7–12 µm, and slightly pitted or not. Alar cells are differentiated, brownish, extending to the costa, with 2–3(–4) layers of cells in the transverse section. Sporophyte not seen.

**Note:** *Dicranum dispersum* is predominantly found in various regions across Europe, (e.g., France, Germany, and Switzerland), Russia, Iran, Mongolia, and North America [39]. In China, it grows on rocky surfaces, associated with *Thuidium cymbifolium* (Dozy & Molk.) Dozy & Molk.

*Dicranum dispersum* has been classified as an endangered species in the Red Lists of Germany and France [40] and assessed as endangered under criteria D in *The IUCN Red List of Threatened Species* [39]. In recent years, new distribution sites of this rare species have been discovered in Europe, Asiatic Russia, and Alaska, U.S.A. [5,41,42], and our study confirms its presence in Qinghai Province, China. To date, the known populations are highly fragmented due to habitat fragmentation, resulting in small, isolated patches that are unable to sustain minimum viable populations and are at risk of entering an extinction vortex [43,44]. Furthermore, moss species, which prefer cold environments, may be more vulnerable to the global warming climate [45,46]. Therefore, we recommend including this rare species on the China Species Red List and advocating for further research into the distribution and biology of high-altitude distributed *Dicranum* species.

**Figure 6:**
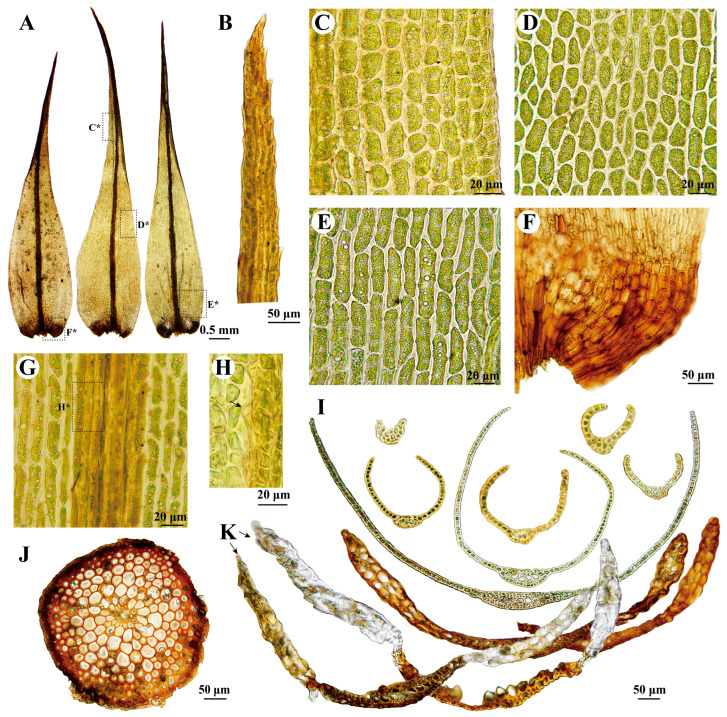
***Dicranum spadiceum*** J.E. Zetterst. (**A**–**G**,**I**,**J**) from *L. Shu & W.Z. Huang 20220817-38* (HSNU); (**H**,**K**) from *M. Sulayman 16887* (XJU). (**A**) Leaves, C*, D*, E*, F* indicates the position of C, D, E, F on the leaf, respectively; (**B**) Leaf apex; (**C**) Upper laminal cells of leaf; (**D**) Middle laminal cells of leaf; (**E**) Basal lamianl cells of leaf; (**F**) Alar cells of leaf; (**G**,**H**) Costa in the upper portion of leaf (dorsal view), H* indicates the position of H, arrow in H shows weakly mammillose; (**I**,**K**) Cross-sections of leaf; (**J**) Cross-section of stem.

The important features of *Dicranum dispersum* include the (1) differentiated dorsal and ventral epidermal layers of costal cells (Figure 4B), (2) leaves that are channeled or keeled in the upper part, appearing V-shaped or tong-like in cross-sectional appearance (Figure 4B), (3) the presence of 2–3(–4) layers of alar cells (Figure 4B), (4) laminal cells that are occasionally or not pitted in the lower portion of the leaf (Figure 4H), (5) long to shortly excurrent costa (Figure 4A), and (6) laminal cells that are bistratose at the margins in the upper portion of the leaf (Figure 4B,E). Additional illustrations can refer to Afonina and Breen [41], Engelmark [47], Otnyukova [48], Ignatov and Ignatova [49], and Lüth [50].

This species can be confused with *D. muehlenbeckii* Bruch & Schimp., *D. spadiceum* J.E.Zetterst., *D. acutifolium*, *D. fuscescens*, *D. brevifolium*, *D. caesium* Mitt., and *D. crispifolium.* The differences between these species have been well discussed by Afonina and Breen [41], Engelmark [47], and Otnyukova and Ochrya [51].

***Dicranum schljakovii*** Ignatova & Tubanova, Arctoa 24(2): 483. 2015 (Figure 2E,F and Figure 5).

**Distribution in China:** inner Mongolia (present report), Xinjiang (present report), new to China.

**Specimens examined: China. Inner Mongolia.** Great Khingan Range, Genhe City, Alongshan Town, Mount Aokelidui (Mount Xianbei), 51°50′38.95′′ N, 122°2′49.08′′ E, 1447 m, on soil, 3 August 2022, *R.L. Zhu* et al. *20220803-314* (HSNU); **Xinjiang**. Altai region, Altai Mt., Dahe tree farm, 47°5′16′′ N, 87°02′24′′ E, 1900 m, on soil, 30 June 1985, *M. Sulayman 1820A* (XJU); Altai region, Borjin County, Kanas National Nature Reserve, 48°41′17.50′′ N, 87°15′12.87′′ E, 3200 m, on decaying wood, 2 July 1998, *M. Sulayman 9861* (XJU); ibid., *M. Sulayman 14158* (XJU); ibid., 48°35′28′′ N, 87°52′47.69′′ E, 2321 m, on stone, 27 September 2016, *M. Sulayman 29199* (XJU); ibid., 48°41′14.42′′ N, 87°14′17.70′′ E, 2225 m, on soil, 10 July 2016, *M. Sulayman 28070* (XJU); Altai region, Altai Mt., Qinghe County, Daqinghe, 46°55′48.46′′ N, 90°23′26.13′′ E, 1860 m, on stone, 16 June 2016, *M.Sulayman 28281* (XJU); ibid., Bianhaizi, 46°49′25.55′′ N, 90°42′43.75′′ E, 2657 m, on stone, 4 August 2015, *M. Sulayman 26705* (XJU).

**Description:** Plants in loose tufts, yellowish to light green. Stems are 2–2.5 cm, matted with white tomentum in the upper part, brownish below. Cross-section of stem is rounded, consisting of 2–3 layers of smaller, obviously thick-walled outer cortical cells, numerous rows of larger and thinner-walled inner cortical cells, and 5–7 rows of central strand cells. Leaves are erect-spreading when wet, loosely appressed and slightly flexuous when dry, 4.5–5.7 × 0.73–0.83 mm, and lanceolate. Leaf margins are subentire, slightly serrulate distally, and unistratose. Costa is ca. 100 µm wide at the leaf base, about 1/7 of leaf width at the base, slightly mammillose in the upper part on abaxial surface, with one row of guide cells in the transverse section, two stereid bands, and differentiated ventral and dorsal epidermis. Leaf laminal is unistratose and smooth. Upper laminal cells are (11–)15–25(–30) × 5–11 µm, irregular in shape, and moderately thick-walled; middle laminal cells are 15–33(–46) × 6–11 µm, rectangular, moderately thick-walled, and slightly porose. Basal laminal cells are elongated–rectangular, 46–80 × 5–13 µm, and pitted. Alar cells are well differentiated, bistratose, brownish, and not extending to the costa, with a group of thin-walled hyaline cells between the alar cells and costa. Setae are 1.0–1.5 cm. Capsules are curved, furrowed, and ca. 2 mm long. Annuli not seen; operculum with a long, straight beak. Spores are 20–25 μm and papillose.

**Note:** In China, *Dicranum schjakovii* grows on soil or decaying wood at altitudes ranging from 773 m to 3200 m. It is usually associated with *Pleurozium schreberi* (Willd. *ex* Brid.) Mitt. and *Ptilidium pulcherrimum* (Weber) Vain. *D. schjakovii* is easily confused with *D. spadiceum* owing to the irregular upper laminal cells in both species, but can be separated by the size of their leaves: *D. schljakovii* has leaves measuring 4.5–5.7 × 0.73–0.83 mm, while *D. spadiceum* has larger leaves measuring 7–8 × 1.3–1.5 mm.

***Dicranum spadiceum*** J.E.Zetterst., Kongl. Svenska Vetensk. Acad. Handl., n.s. 5(10): 20. 1865 (Figure 2G,H and Figure 6).

**Distribution in China:** Qinghai (present report), Xinjiang (present report), new to China.

**Specimens examined: China. Xinjiang.** Urumqi City, Urumqi County, Mount Nanshan, Xiaoquzi forestry farm, 43°26′63′′ N, 87°05′22′′ E, 2420 m, on soil, 2 July 2011, *Horyat Abliz 450* (XJU); Xinjiang, Altai region, Altai city, Xiaodonggou Forest Park, 48°00′20.4′′ N, 88°18′33.5′′ E, 1904 m, on rock, 7 August 2011, *M. Sulayman 16887* (XJU); Xinjiang, Mount Altai, Qiinggil County, Qibahalegai, 47°04′14.00′′ N, 90°13′59.37′′ E, 1600 m, on decaying wood, 2 August 2015, *M. Sulayman 26582* (XJU); Qoqak, the Barluk Mountain National Nature Reserve, 45°47′58′′ N, 82°59′50′′ E, 1781 m, on soil, 22 July 2022, *M. Sulayman 37449* (XJU). **Qinghai.** Haidong City, Huzhutuzu Autonomous County, Huzhubeishan National Forest Park, near the Shengmutianchi, 36°53′0.94″N, 102°19′39.81″E, 3737 m, on soil, 17 August 2022, *L. Shu & W.Z. Huang 20220817-34* (HSNU), *L. Shu & W.Z. Huang 20220817-38* (HSNU), *L. Shu & W.Z. Huang 20220817-39* (HSNU).

**Description:** Plants in loose tufts, light brownish green. Stems are 3–5 cm and moderately tomentose. Cross-section of stem is rounded, consisting of 2–4 layers of smaller, obviously thick-walled outer cortical cells, numerous rows of larger and thinner-walled inner cortical cells, and 5–7 rows of central strand cells. Leaves are straight and erect when wet, loosely appressed when dry, 7–8 × 0.13–0.15 mm, gradually to abruptly narrowed into long and narrow tubular acumen from the ovate base, semicircular in the transverse section. Leaf margins are subentire, slightly serrulate distally, and unistratose. Costa is ca. 120 µm wide at the leaf base, about 1/13 to 1/10 of leaf width at the base, smooth or weakly mammillose in the upper part on the abaxial surface, with one row of guide cells in transverse section, two stereid bands, and differentiated ventral and dorsal epidermis. Leaf lamina is unistratose and smooth. Upper and middle laminal cells are 20–41 × 9–13 µm, irregular in shape, moderately thick-walled, and slightly porose. Basal laminal cells are elongated–rectangular, 45–85 × 8–12 µm, and pitted. Alar cells are well differentiated, 2-4-stratose, brownish, and hyaline, with few or no thin-walled cells between the costa and alar groups. Sporophyte not seen.

**Note:** This species is widely distributed in Europe [2], North America [3], and Russia [5]. In China, we discovered this species in an alpine meadow in Qinghai, along with three specimens from Xinjiang. It grows on soil, rock, or decaying wood, from 1600 m to 3737 m, usually associated with *Cirriphyllum cirrosum* (Schwägr.) Grout, *Sanionia uncinata* (Hedw.) Loeske, and *Distichium capillaceum* (Hedw.) Bruch & Schimp.

According to the description and illustration of the lectotype specimen of *Dicranum spadiceum* by Ignatova et al. [5], only two layers of alar cells are present in this species. This characteristic was also noted and confirmed by Hedenäs and Bisang [2], Ireland Jr. [3], Ignatov and Ignatova [49], and Lüth [50]. In our samples, three specimens from Xinjiang also exhibit bistratose alar cells (Figure 6K), while two specimens from Qinghai show partially 3–4-layer alar cells (Figure 6I). Our phylogenetic analyses indicate that these two intriguing samples from Qinghai are highly nested within the other samples (BS_ML_ = 99, PP_BI_ = 1; Figure 1), suggesting that they belong to *D. spadiceum*. We propose that this characteristic may be influenced by environmental factors, as the two specimens from Qinghai were found at high altitudes of 3737 m, whereas specimens from Xinjiang (this study), Europe, and Asian Russia were found at lower altitudes ranging from 704 m to 2430 m [5]. The differences between *D. spadiceum* and *D. schljakovii* are discussed under the latter species.

### 2.3. Taxonomy Treatment

***Dicranoloma fragile*** Broth. Nat. Pflanzenfam. (ed. 2) 10: 209. 1924.

**≡***Dicranum psathyrum* Klazenga, J. Hattori Bot. Lab. 87: 118. 1999. **Type:** Nepal. In Nepaul legit Hon. D. Gardner, et ad J. Banks Baronetum communicavit Gul. Wallich (BM). ***syn. nov.***

**Distribution in China:** *Dicranoloma fragile* is common in the south of China [33,34,35,36], including Anhui, Fujian, Guangdong, Guangxi, Guizhou, Hainan, Hunan, Sichuan, Xizang, Yunnan, and Zhejiang [33,34].

**Notes:** This species was initially described as *Dicranum fragile* Hook., nom. illeg., by Hooker [52]. Later, Brotherus [53] formally published the species as *Dicranoloma fragile* Broth. However, Klazenga [54] moved this species to the genus *Dicranum* due to the absence of limbidium and differences in the transverse section of the costa from all *Dicranoloma* species. Nevertheless, the epithet “fragile” was already in use for *Dicranum fragile* Brid. (≡*Campylopus fragile* (Brid.) Bruch & Schimp.) at that time. Consequently, Klazenga [54] proposed using “psathyrum” as an alternative.

According to our phylogenetic analysis, *Dicranum psathyrum* was found deeply nested within the genus *Dicranoloma* (BS_ML_ = 99, PP_BI_ = 0.93) (Figure 1). Consequently, Klazenga’s [54] taxonomic classification should be rejected, the taxonomic status of *Dicranoloma fragile* needs to be reinstated, and *Dicranum psathyrum* should be treated as a synonym of *Dicranoloma fragile*.

### 2.4. Chinese Dicranum Species and Annotation

***Dicranum assamicum*** Dixon, J. Bombay Nat. Hist. Soc. 39: 774. 1937.

**Distribution in China:** Distributed only in the Himalayan region in China [35,36,55], including Chongqing, Sichuan [33,34], Yunnan [56], and Xizang [30,33,34].

**Note:** *Dicranum assamicum* may be confused with *D. japonicum* Mitt. because both species possess 2–4 layers of alar cells. However, *D. assamicum* can be well distinguished by the serrated costa on the upper portion of the leaf, straight capsules, smooth peristome teeth above, and the absence of minute and longitudinal point striations below [30,56,57], whereas *D. japonicum* is characterized by 2–3(–4) serrated ridges on the upper portion of the costa, slightly curved capsules, densely papillose peristome teeth above, and minute and longitudinal point striations below [58].

***Dicranum baicalense*** Tubanova, Arctoa 31(2): 147. 2022.

**Distribution in China**: inner Mongolia [38].

**Note:** This species has been known from Russia (southern Siberia and the southern part of the Russian Far East) [10] and China [38].

*Dicranum baicalense* may be mistakenly identified as *D. leioneuron* Kindb., *D. nipponense* Besch., and *D. japonicum* in China. However, they can be distinguished by the following characteristics: (1) *D. leioneuron* is characterized by a straight capsule, while *D. baicalense* has an arcuate capsule; (2) *D. japonicum* features alar cells that are 2–4-layered, with the capsule becoming striated when dry; in contrast, *D. baicalense* has 2-layered alar cells and a smooth capsule when dry; (3) *D. nipponense* has distal leaf cells measuring 50–80 µm, with short cells above the alar cells extending upwards along the leaf margins, whereas *D. baicalense* has distal leaf cells measuring 65–100 µm and lacks short cells above the alar cells.

***Dicranum bardunovii*** Tubanova & Ignatova, Arctoa 20: 185. 2011. (Figure 2A,B and Figure 3)

**Note:** New to China (present study).

***Dicranum bonjeanii*** De Not., Elenc. Musch.: 29. 1837.

**Distribution in China:** Heilongjiang [33,34], Jilin [33,34], inner Mongolia [33,34,59], Shaanxi [60], and Yunnan [38].

**Note:** *Dicranum bonjeanii* can be recognized by its elongated–rectangular upper cells, weakly to strongly transversely undulate upper leaf portions, and comparable orientation of apical and lower leaves. This species could only be confused with *D. polysetum* Sw. in China, and the differences between them are discussed under *D. polysetum*.

***Dicranum cheoi*** E.B.Bartram, Ann. Bryol. 8: 8. 1936.

**Distribution in China:** Endemic to China, known only from Guizhou [33,34,61], and Xizang [33,34].

**Note:** *Dicranum cheoi* may be confused with *D. fuscescens, D. hamulosum* Mitt., *D. bardunovii*, and *D. muehlenbeckii* Bruch & Schimp. However, the epidermal cells on the ventral side of the costa are not differentiated in *D. fuscescens*, *D. hamulosum*, and *D. bardunovii*, but well differentiated in *D. cheoi*. In addition, the upper leaf lamina is bistratose along the margins in *D. cheoi*, but unistratose in *D. muehlenbeckii.*

***Dicranum crispifolium*** Müll.Hal., Bot. Zeitung (Berlin) 22: 349. 1864.

**Distribution in China:** Sichuan [33,34], Yunnan [33,34], Xizang [33,34].

**Note:** Both *Dicranum drummondii* and *D. dispersum* exhibit multilayered alar cells, and quadrate or rectangular laminal cells in the upper portion of the leaf, resembling those of *D. crispifolium*. However, epidermal cells on the ventral side of the costa are differentiated in *D. dispersum* but not differentiated in *D. crispifolium*. Differences between *D. crispifolium* and *D. drummondii* are discussed under the latter species.

***Dicranum dispersum*** Engelmark, Stuttgarter Beitr. Naturk., A 592: 4. 1999 (Figure 2C,D and Figure 4).

**Note:** New to China (present study).

***Dicranum drummondii*** Müll.Hal., Syn. Musc. Frond. 1: 356. 1848.

**Distribution in China:** Mainly distributed in the northeast and southwest of China [35,36,55], including Guizhou [33,34,61], Heilongjiang [28,59], inner Mongolia [59], Jilin [28,33,34], Shaanxi [33,34], Sichuan [33,34], and Xizang [33,34].

**Note:** *Dicranum drummondii*, a member of the large-sized *Dicranum* species, is frequently confused with *D. crispifolium* in China. These two species share 3–5-layered alar cells and papillose laminal cells on the dorsal surface in the upper portion of the leaf. However, *D. drummondii* can be easily separated by its unistratose laminal cells along the margins, while they are bistratose in *D. crispifolium*. In addition, the upper leaf laminal cells are rugose in *D. drummondii*, contrasting with the non-rugose leaves of *D. crispifolium*.

***Dicranum elongatum*** Schleich. *ex* Schwägr., Sp. Musc. Frond., Suppl. 1, 1: 171. 1811.

**Distribution in China:** Guizhou [61], Hebei [33,34], inner Mongolia [33,34,59], Jilin [33,34], Liaoning [34], Sichuan [33,34], Xinjiang [34], Yunnan [33,34,56].

**Note:** *Dicranum elongatum* is closely related to *D. setifolium* Cradot and *D. groenlandicum*, but can be distinguished by the presence of short, smooth laminal cells on the upper portion of the leaf. In contrast, *D. setifolium* exhibits elongated–rhomboidal cells, and *D. groenlandicum* exhibits long, porose cells throughout the leaf.

***Dicranum flagellare*** Hedw., Sp. Musc. Frond.: 130. 1801.

**Distribution in China:** Common in the northeast and southwest of China [35,36,55], including Guangxi, Heilongjiang [33,34], Hubei, inner Mongolia [59], Jilin [33,34], Shandong [62], Taiwan, Xizang [30], and Yunnan (present report).

**Note:** *Dicranum flagellare* is easily recognized by its small size and universal occurrence of numerous flagellae that arise in the upper leaf axils. This species bears a resemblance to *D. mayrii* Broth. and *D. ignatovii* Tubanova & Fedosov in terms of flagelliform branchlets but differs from *D. ignatovii* by its unistratose alar cells (in contrast to the bistratose alar cells in *D. ignatovii*) and differs from *D. mayrii* by its almost smooth laminal cells and costa in the distal portion of leaf (which are sharply and densely scabrose in *D. mayri*).

***Dicranum fragilifolium*** Lindb., Öfvers. Kongl. Vetensk.-Akad. Förh. 14(4): 125. 1857.

**Distribution in China:** Common in the north and southwest of China [35,36,55], including Chongqing, Guizhou [61], Heilongjiang [28,33], Hubei, inner Mongolia [33], Jilin [28], Shaanxi, Sichuan, Taiwan [33], Yunnan [56], Xinjiang [63], and Zhejiang [64].

**Note:** *Dicranum fragilifolium* is characterized by its straight and erect, stiff, and fragile leaves. This species differs from *D. hakkodense* Cardot and *Dicranoloma fragile*, with few blunt teeth at the apices (in contrast to sharply denticulate or serrated in *Dicranum hakkodense* and *Dicranoloma fragile*). Furthermore, it can be distinguished from *Dicranum hengduanense* by the distinct stereid bands present in the lower part of its costa.

***Dicranum fulvum*** Hook., Musci Exot. 2: 149. 1819.

**Distribution in China:** Common in the northeast and southwest of China [35,36,55], including Chongqing [65], Guizhou [33,34], Heilongjiang [28], Jilin [26], Liaoning [26], and Yunnan [56].

**Note:** *Dicranum fulvum* can be distinguished from all Chinese *Dicranum* species by its larger proportion of bistratose leaf lamina, except for *D. shennongjiaense* and *D. majus* Turner. However, laminal cells in the upper leaf portion are regularly quadrate to short rectangular in *D. fulvum*, contrasting with the rectangular and elongated cells in *D. shennongjiaense* and *D. majus*.

***Dicranum fuscescens*** Turner, Muscol. Hibern. Spic. 60. pl. 5: f. 1804.

**Distribution in China:** Guizhou [33,34], Heilongjiang [33,34], Jilin [33,34], Liaoning [33,34], inner Mongolia [33,34], Shaanxi [34], Sichuan [34], Xizang [33,34], and Zhejiang [66].

**Note:** *Dicranum fuscescens* might be confused with *D. muehlenbeckii, D. cheoi*, and *D. hamulosum*, and the differences are annotated under the latter species, respectively.

***Dicranum groenlandicum*** Brid., Muscol. Recent. Suppl. 4: 68. 1819.

**Distribution in China:** Mainly distributed in the north and southwest of China [35,36,55], including Heilongjiang [33,34], inner Mongolia [33,34,59], Jilin [33,34], Xinjiang [63], Xizang [30], and Yunnan [33,34].

**Note:** *Dicranum groenlandicum*, *D. elongatum*, *D. himalayanum* Mitt., and *D. setifolium* are closely related and can be easily confused; the distinctions among them are annotated under the latter three species, respectively.

***Dicranum hakkodense*** Cardot, Bull. Herb. Boissier, sér. 2, 7: 714. 1907.

Distribution in China: Sichuan [6].

**Note:** *Dicranum hakkodense* has long been classified as *D. viride* var. *hakkodense* (Cardot) Takaki, but was subsequently reclassified as a distinct species based on molecular evidence by Ignatova and Fedosov [6]. The presence of sharply denticulate leaf apices serves as a key feature for distinguishing between *D. hakkodense* and *D. viride*; while the former exhibits sharp denticulation apices, the latter has only a few blunt teeth or entirely smooth apices. Variations in comparison to *D. fragilifolium* and *D. hengduanense* are individually discussed within the context of the latter species.

***Dicranum hamulosum*** Mitt., Trans. Linn. Soc. London, Bot. 3: 156. 1891.

**Distribution in China:** Guangxi [34], Hainan [34], Heilongjiang [26], Jilin [33,34], Liaoning [26], Sichuan [34], Taiwan [33,34], Xizang [34], Yunnan [34], and Zhejiang [33,34].

**Note:** *Dicranum hamulosum* might be confused with *D. cheoi* and *D. fuscescens*, but can be distinguished by the unistratose alar cells, contrasting with the bistratose alar cells found in *D. cheoi* and *D. fuscescens*. In addition, the epidermal cells on the ventral side of the costa are undifferentiated in *D. hamulosum* but show differentiation in *D. cheoi.*

***Dicranum hengduanense*** W.Z.Huang & R.L.Zhu, Bryologist 126(2): 229. 2023.

**Distribution in China**: Endemic to China. At present, *Dicranum hengduanense* is known only in two localities in Yunnan province, southwestern China [7].

**Note:** *Dicranum hengduanensis* is one of the *Dicranum* species with fragile leaves. In contrast to *D. hakkodense* and *D. psathyrum* (**≡***Dicranoloma fragile*), this species exhibits entirely smooth leaf apices instead of sharply denticulate ones. Differences from *D. fragilifolium* are discussed under the latter species. In addition, *D. hengduanense* can be distinguished from other *Dicranum* species with delicate leaves by the absence of stereid bands and the presence of only one layer of cells above and below the guide cells [7].

***Dicranum himalayanum*** Mitt., J. Proc. Linn. Soc., Bot., Suppl. 1: 14. 1859.

**Distribution in China:** Mainly distributed in the southwest of China [35,36,55], including Sichuan, Xizang, and Yunnan [30,33,34,59].

**Note:** *Dicranum himalayanum* can be confused with *D. groenlandicum* due to the smooth leaf margin and prosenchymatous and porose laminal cells in the upper portion of the leaf. However, these two species differ in their growth patterns: *D. himalayanum* forms loose tufts with branching and possesses 2–3-layered alar cells, whereas *D. groenlandicum* typically creates very dense cushions with minimal or no branching and has unistratose or occasionally bistratose alar cells [30,34].

***Dicranum japonicum*** Mitt., Trans. Linn. Soc. London, Bot. 3: 155. 1891.

**Distribution in China:** Widely distributed in China [35,36,55].

**Note:** *Dicranum japonicum* may be confused with *D. baicalense*, *D. bonjeanii*, *D. lorifolium*, *D. nipponense*, and *D. scoparium* in China. In its typical forms, *D. japonicum* presents 2–4 layers of alar cells [10]; however, some specimens may exhibit only 2-stratose alar cells [10,58]. *Dicranum japonicum* can be distinguished by the combination of the following characteristics: (1) inclined and asymmetrical capsules, (2) costa with two lamellae or ridges on its back, (3) leaves 8–9(–11) mm long, being about 10 times as long as they are wide, and (4) the absence of short cells above the alar cells. In addition, confusion between *D. japonicum* and *D. assamicum* is possible, with distinct characteristics detailed under *D. assamicum*.

***Dicranum kashmirense*** Broth., Acta Soc. Sci. Fenn. 24(2): 9. 1899.

**Distribution in China:** Mainly distributed in the south of China [35,36,55], including Chongqing, Guangxi [33,34,59], Guizhou [61], Hubei, Hunan [33,34,59], Jiangxi [67], Sichuan [33,34,59], and Yunnan [56].

**Note:** *Dicranum kashmirense* may be confused with *D. scoparium* but can be distinguished by the unistratose alar cells in *D. kashmirense*, while they are bistratose in *D. scoparium*.

***Dicranum leiodontum*** Cardot, Bull. Herb. Boissier, sér. 2, 7: 714. 1907.

**Distribution in China:** Jilin, Xinjiang, and Xizang [31,33,34,35,36,55,63].

**Note:** *Dicranum leiodontum* may be confused only with *D. papillidens*, with the distinctions between them detailed under the latter species.

***Dicranum linzianum*** C.Gao, Acta Phytotax. Sin. 17: 115. 1979.

**Distribution in China:** Endemic to China, known only from Xizang [33,34,35,36,55].

**Note:** *Dicranum linzianum* may be confused exclusively with *D. spurium* due to both having ovate–lanceolate leaves, and the distinguishing characteristics are discussed under the latter species.

***Dicranum lorifolium*** Mitt., J. Proc. Linn. Soc., Bot., Suppl. 1: 15. 1859.

**Distribution in China:** Mainly distributed in the south of China [35,36,55], including Chongqing, Fujian [33,34], Gansu [33,34], Guizhou [33,34], Jiangxi [67], Taiwan, Xizang [33,34], Yunnan [33,34], and Zhejiang [68].

**Note:** *Dicranum lorifolium* may be confused with *D. assamicum*, *D. baicalense*, *D. bonjeanii*, *D. japonicum*, *D. nipponense*, and *D. scoparium* in China, and it is difficult to distinguish them based solely on gametophyte. However, *D. lorifolium* can be well distinguished by the following unique combination of characteristics: (1) bistratose alar cells, (2) costa above with two lamellae or ridges on its back, (3) the presence of short cells above the alar cells, extending upwards along the leaf margins, and (4) always erect capsules.

***Dicranum majus*** Turner, Muscol. Hibern. Spic.: 58. 1804.

**Distribution in China:** Widely distributed in China [35,36,66].

**Note:** *Dicranum majus* is one of the largest members of the genus when well developed. This is a very variable species [2,69], in terms of leaf curvature and length, the upper leaf lamina being partly bistratose or not, and the upper laminal cells spinosely projecting or not [2]. *D. majus* may be confused with *D. scoparium*, but *D. scoparium* has four distinct ridges on the upper back of the costa rather than furrows and serrations in *D. majus*. In addition, setae generally aggregate in *D. majus* but are solitary in *D. scoparium* [69].

***Dicranum mayrii*** Broth., Hedwigia 38: 207. 1899.

**Distribution in China:** Chongqing [65], Heilongjiang [33,34], and Taiwan [33,34].

**Note:** *Dicranum mayrii* is easily recognized by its universal occurrence of numerous flagellae and mammillose upper laminal cells on the dorsal side. This species could only be confused with *D. flagellare* in China, and differences between these two species are discussed under *D. flagellare*.

***Dicranum montanum*** Hedw., Sp. Musc. Frond.: 143. 1801.

**Distribution in China:** Widely distributed in China [35,36,55], including Guizhou [61], Hainan [33,34], Hebei [33,34], Heilongjiang [33,34], Hubei, inner Mongolia [33,34], Jilin [33,34], Liaoning, Shaanxi [60], Sichuan [60], Xizang [30,33,34], Yunnan [56,60], and Zhejiang [60].

**Note:** *Dicranum montanum* is one of the smallest species within the genus in China and is easily recognized because it looks more like *Dicranoweisia cirrata* (Hedw.) Lindb. than a *Dicranum* species. In addition, another aid in its identification is the occasional presence of small, delicate, clustered branchlets with linear leaves that are readily detachable (probably a means of asexual reproduction like *Brothera leana* (Sull.) Müll.Hal.), which occur near the stem apices.

***Dicranum muehlenbeckii*** Bruch & Schimp., Bryol. Eur. 1: 142. 1847.

**Distribution in China:** Guizhou [28,61], Heilongjiang [28], inner Mongolia [34], Jilin [33,34], Liaoning [28], Shaanxi [55], Sichuan [33,34], Taiwan [55], Xinjiang [28,30,63], Xizang [33,34], Yunnan [56], and Zhejiang [33,34].

**Note:** *Dicraunm muehlenbeckii* may be confused with *D. fuscescens*; however, the epidermal cells on the ventral side of the costa are not differentiated in *D. fuscescens*, whereas they are well differentiated in *D. muehlenbeckii.*

***Dicranum nipponense*** Besch., Ann. Sci. Nat., Bot., sér. 7, 17: 332. 1893.

**Distribution in China:** Widely distributed in China [35,36,55].

**Note:** *Dicranum nipponense* may be confused with *D. assamicum*, *D. baicalense*, *D. bonjeanii*, *D. japonicum*, *D. lorifolium,* and *D. scoparium* in China. However, it can be distinguished by the following combination of characteristics: (1) bistratose alar cells, (2) costa above with two lamellae or ridges on its back, (3) the presence of short cells above the alar cells, extending upwards along the leaf margins, (4) capsules inclined and asymmetrical, (5) dark green plants, and (6) leaves 3–7 mm long.

***Dicranum papillidens*** Broth., Sitzungsber. Akad. Wiss. Wien, Math.-Naturwiss. Kl., Abt. 1, 133: 561. 1924.

**Distribution in China:** Endemic to China, known only from Sichuan [33,34,55].

**Note:** *Dicranum papillidens* was first reported by Brotherus [20] in 1924 and has remained elusive for almost a century. This species is characterized by (1) unistratose alar cells, (2) quadrate or short rectangular cells in the upper leaf, (3) a smooth abaxial surface of the upper laminal cells and costa, (4) being strongly porose in the basal and middle leaf cells, and (4) having densely papillose peristome teeth throughout. *Dicranum papillidens* can be easily separated from *D. leiodontum* by its strongly porose basal and middle laminal cells as well as its densely papillose peristome teeth.

***Dicranum polysetum*** Sw., Monthly Rev. 34: 538. 1801.

**Distribution in China:** Mainly distributed in the north and southwest of China [35,36,55], including Heilongjiang [33,34], Henna [69], inner Mongolia [33,34,59], Jilin [33,34], Xinjiang [63], Xizang [33,34], and Yunnan.

**Note:** *Dicranum polysetum* is readily distinguishable in the field due to its distinctive leaf orientation, with erect uppermost leaves and spreading lower leaves, as well as strongly undulate leaves. This species can only be confused with *D. bonjeanii*; however, the orientation of the apical leaves and leaves further down are not clearly different in the latter species.

***Dicranum rectifolium*** Müll.Hal., Nuovo Giorn. Bot. Ital., n.s. 3: 98. 1896.

**Distribution in China:** Endemic to China, known only from Shaanxi [15].

**Note:** This species was initially reported by Müller in Shaanxi [15] but has not been rediscovered for over 125 years. Regrettably, there is no available information regarding illustrations or descriptions of this species. This species was classified as an insufficiently known species in the earlier Chinese *Checklist of the Mosses* [70].

***Dicranum schljakovii*** Ignatova & Tubanova, Arctoa 24(2): 483. 2015 (Figure 2E,F and Figure 5).

**Note:** New to China (present study).

***Dicranum scoparium*** Hedw., Sp. Musc. Frond. 126. 1801.

**Distribution in China:** Widely distributed in China [35,36,55].

**Note:** *Dicranum scoparium* is one of the most variable *Dicranum* species, with both straight and falcate leaves commonly observed, and the presence of both dwarf and large male plants. In China, this species can be distinguished from other members of the *D. scoparium* complex (including *D. baicalense*, *D. bonjeanii*, *D. japonicum*, *D. lorifolium*, *D. nipponense*, and *D. polysetum*) by the four ridges on the dorsal surface of its costa, as opposed to the typical two ridges found in other species.

***Dicranum scottianum*** Turner, Muscol. Hibern. Spic.: 75. 1804.

**Distribution in China:** Heilongjiang [28], inner Mongolia [59], Jilin [28], and Taiwan (present study).

**Note:** *Dicranum scottianum* is characterized by (1) its asymmetrical leaves, with one side of the leaf being wider than the other, (2) the difference in the angle of insertion of the lamina with respect to the costa, (3) the laminal cells that are thick-walled, and (4) the wide and deep costa that has two bands of stereids. In the only specimen we examined from Taiwan, the margins are dentate in the upper part of the leaf, which can be referrable to *D. canariense* Hampe ex Müll.Hal. [2]; however, Price et al. [71] synonymized it under *D. scottianum* in 2016.

***Dicranum setifolium*** Cardot, Bull. Herb. Boissier, sér. 2, 7: 714. 1907.

**Distribution in China:** Jilin and Sichuan [33,34,35,36,55].

**Note:** *Dicranum setifolium* shows a close relation to *D. elongatum* and *D. groenlandicum* but is distinguished from the latter two species by the characteristics of its upper and middle laminal cells. In *D. groenlandicum*, these cells are porose, while in *D. setifolium*, they are smooth. The differences between *D. setifolium* and *D. elongatum* are discussed under the latter species.

***Dicranum shennongjiaense*** W.Z.Huang & R.L.Zhu

Distribution in China: Hubei [38].

**Note:** Refer to Huang et al. [38] for details.

***Dicranum spadiceum*** J.E.Zetterst., Kongl. Svenska Vetensk. Acad. Handl., n.s. 5(10): 20. 1865 (Figure 2G,H and Figure 6).

**Note:** New to China (present study).

***Dicranum spurium*** Hedw., Sp. Musc. Frond.: 141. 1801.

**Distribution in China:** Mainly distributed in the northeast of China [35,36,55], including Heilongjiang [28], inner Mongolia [33,34,59], and Jilin [33,34].

**Note:** *Dicranum spurium* may be confused with *D. linzianum* in China as both species exhibit ovate–lanceolate leaves (relatively broad basal portion, becoming suddenly narrow to a short acumen). However, these species can be differentiated by the upper laminal cells: those of *D. spurium* are irregular, triangular, or quadrate, with prorulae on the dorsal surface, whereas *D. linzianum* has oval and smooth upper laminal cells.

***Dicranum undulatum*** Schrad. *ex* Brid., J. Bot. (Schrader) 1800. 1(2): 294. 1801.

**Distribution in China:** Mainly distributed in the northeast of China [35,36,55], including Heilongjiang [33,34], inner Mongolia [33,34], Jilin [33,34], Liaoning [28], and Xinjiang [28].

**Note:** *Dicranum undulatum* may be confused with *D. polysetum* and *D. bonjeanii* in China due to their similarly transversely undulating leaves but can be distinguished by the irregular oval shape of the upper laminal cells, which differ from the elongated–rectangular cells found in the latter two species.

### 2.5. Excluded Species

***Dicranum brevifolium*** (Lindb.) Lindb., Musci Scand.: 24. 1879.

**Note:** Sulayman et al. [72] reported the presence of this species in the Altai Mountains, Xinjiang. Although we have not seen the voucher specimen directly, based on their illustrations, it is clearly an incorrect identification: (1) the transverse section of the upper leaf looks like a pair of tongs in *D. brevifolium* [2,12,47], but it looks more or less like a circular outline in their illustration; (2) the ventral epidermal layer of cells, which are usually poorly differentiated in *D. brevifolium* [2,8,47], are distinctly differentiated in their illustration. In addition, (3) while the leaf lamina is bistratose along the margins in *D. brevifolium*, it is unistratose in their illustrations. In conclusion, *D. brevifolium* should be excluded from the moss flora of China.

***Dicranum viride*** (Sull. & Lesq.) Lindb., Hedwigia **2**(11): 70. 1863.

**Note:** When Ignatova and Fedosov revised *Dicranum* species with fragile leaves in Russia [6], they found that all records of *D. viride* in the Russian Far East actually belong to *D. hakkodense*, and its occurrence in Japan, Korea, and China, as well as in Alaska seems to be doubtful. In China, this species has been recorded in Chongqing [35,36], Guizhou [33,34,61], Hubei [73], Sichuan [33,34], Xinjiang [74], and Yunnan [33,34,56]. However, all illustrations and descriptions of this species in China [33,34,56,61], characterized by serrated leaf apices and elongated–rectangular basal laminal cells, align with *D. hakkodense.* In addition, our examination of specimens previously labeled as *D. viride* in China, including *M. Zang-5276*, *L.S. Wang-879*, and *X.J. Li-4329* from Yunnan (KUN), *G.L. Zheng 231* from Hubei (CCNU), *M. Sulayman 13008, 25902, 17145* from Xinjiang (XJU), *Q. Gao* et al. *3186* from inner Mongolia (PE), *Huang & Li 2248* from Shaanxi (PE), *Y. Liu 320* from Sichuan (PE), and *F081116, L. He 050912425* from Guizhou (GACP), revealed that none of them correspond to *D. viride*.

### 2.6. Key to Dicranum Species in China

Note: *Dicranum rectifolium* is not included because we have no information, illustrations, or descriptions of this species, which was merely relisted without additional specimens in a checklist from 1896 and was listed as an insufficiently known species in China [70].

1. Leaves stiff and fragile, upper leaf portions very narrow, and tips frequently broken….2
1. Leaves usually crisped or falcate, tips mostly present...........................................................6
2. Leaf apices entirely smooth or with few blunt teeth..............................................................3
2. Leaf apices sharply denticulate or serrulate............................................................................4
3. Costa in lower portion of the leaf with stereid bands, sometimes weak, with up to 2–3(–4) layers of cells above and below guide cells...........................................*D. fragilifolium* Lindb.
3. Costa in lower portion of the leaf lacking stereid bands, with up to (0–)1–2 layers of cells above and below guide cells.......................................*D. hengduanense* W.Z.Huang & R.L.Zhu
4. Margin serrated in the upper 1/2–3/4(–5/6), entirely smooth below. Basal laminal cells elongated–rectangular, 30–90(–120) µm long…………………………………...*Dicranoloma fragile* Broth. (≡ *Dicranum psathyrum* Klazenga)
4. Margin serrated only near apex. Basal laminal cells short rectangular, 20–50 µm long….5
5. Leaves only occasionally fragile, crisped when dry; basal laminal cells quadrate to short rectangular, 20–35 µm long; costa broader, occupying 1/3 or more of the total leaf base width; leaf lamina mostly 2-stratose above.........................................*D. fulvum* Hook. (in part)
5. Leaves moderately fragile, straight, or slightly falcate–secund when dry; basal laminal cells elongated–rectangular, 25–50 µm long; costa narrower, occupying less than 1/3 the leaf base width; leaf lamina mostly 1-stratose above...............................*D. hakkodense* Cardot
6. Plant with flagelliform branchlets (rigid and terete branches with appressed leaves) in the distal leaf axils...........................................................................................................................7
6. Plant without flagelliform branchlets......................................................................................8
7. Upper laminal cells smooth on dorsal surface...........................................*D. flagellare* Hedw.
7. Upper laminal cells mammillose on dorsal surface.......................................*.D. mayrii* Broth.
8. Laminal cells irregularly bistratose in the upper portion of the leaf…………...………..…9
8. Laminal cells unistratose in the upper portion of the leaf, or bistratose only along margins….............................................................................................................................................11
9. Laminal cells in the upper leaf portion are regularly quadrate to short rectangular…………………………….....................….......…...………….*D. fulvum* Hook. (in part)
9. Laminal cells in the upper leaf portion are rectangular and elongate….…….….……….10
10. Costa with 2 rows of guide cells……………………………....……….…...*D. majus* Turner
10. Costa with 1 row of guide cells…………..……*D. shennongjiaense* W.Z.Huang & R.L.Zhu
11. Cells in upper leaf prosenchymatous and porose..............................................................12
11. Cells in upper leaf quadrate or rectangular, rarely elongated–rectangular, with or without pores........................................................................................................................................23
12. Leaves straight, in upper part more or less tubular, apex rounded, margin entirely smooth throughout or sometimes with a few obtuse denticles near apex.............................13
12. Leaves falcate or straight, in upper part flat or channeled, apex acuminate, margin above finely to coarsely denticulate...........................................................................................14
13. Plants in loose tufts, alar cells 2–3-layered............................*D. himalayanum* Mitt. (in part)
13. Plants in dense cushions, alar cells unistratose or rarely bistratose..........................................................................................................................................................*.D. groenlandicum* Brid.
14. Costa above with at least four lamellae or numerous ridges or furrows on back (at least in some leaves).................................................................................................*D. scoparium* Hedw.
14. Costa above with two lamellae, or ridges, or smooth on back...........................................15
15. Leaf lamina transversely undulate.......................................................................................16
15. Leaf lamina not undulate.......................................................................................................17
16. Leaf lamina strongly transversely undulate; margin in upper part spinosely denticulate or dentate. Apical leaves erect, leaves below spreading...........................*D. polysetum* Sw.
16. Leaf lamina more or less strongly transversely undulate (at least in some leaves); margin obtusely to sharply denticulate. Orientation of apical leaves and leaves further down not clearly different.........................................................................................*D. bonjeanii* De Not
17. Alar cells unistratose or bistratose.......................................................................................18
17. Alar cells 2–4-layered.............................................................................................................42
18. Alar cells unistratose...............................................................................*D. kashmirense* Broth.
18. Alar cells bistratose.................................................................................................................19
19. Costa above smooth on back...................................................*D. himalayanum* Mitt. (in part)
19. Costa above with two lamellae or ridges on back...............................................................20
20. Capsules straight...........................................................................................*D. lorifolium* Mitt.
20. Capsules arcuate.....................................................................................................................21
21. Leaves 8–9(–11) mm long..............................................................*D. japonicum* Mitt. (in part)
21. Leaves 3–7.5 mm long............................................................................................................22
22. Plants dark green, presence of short cells above the alar cells, extending upwards along the leaf margins...............................................................................................*D. nipponense* Besch.
22. Plants yellowish to light green, no presence of short cells above the alar cells.....................................................................................................................................*D. baicalense* Tubanova
23. Upper leaf lamina in at least some leaves rugose, or more or less transversely undulate (dry and wet).................................................................................................................................24
23. Upper leaf lamina neither rugose nor undulate..................................................................26
24. Leaf lamina transversely undulate, in upper part with smooth or lowly mammillose cells; basal leaf portion lanceolate, leaf apex more or less obtuse.............................................................................................................................................*D. undulatum* Schrad. *ex* Brid.
24. Leaf lamina rugose, in upper leaf on back with spine-like or conically projecting cells; basal leaf portion ovate, leaf apex acuminate............................................................................25
25. Upper leaf laminal cells irregularly quadrate, walls esinuose; leaves gradually narrowed to long, falcate acumen...............................................................*D. drummondii* Müll.Hal.
25. Upper leaf laminal cells very irregular, triangular, quadrate, with sinuose walls; leaves suddenly narrowed to short acumen...............................................................*D. spurium* Hedw.
26. Alar cells unistratose..............................................................................................................27
26. Alar cells bistratose or multilayered.....................................................................................30
27. Upper laminal cells mammillose on abaxial surface..........................................................28
27. Upper laminal cells smooth on abaxial surface...................................................................29
28. Plant small, leaf laminal cells unistratose..............................................*D. montanum* Hedw.
28. Plant size variable, leaf laminal cells bistratose along margins.............*D. hamulosum* Mitt.
29. Peristome teeth densely papillose throughout......................................*D. papillidens* Broth.
29. Peristome teeth smooth above, faintly vertically striated below......*D. leiodontum* Cardot
30. Upper leaf laminal cells bistratose along margins..............................................................31
30. Leaf laminal cells unistratose................................................................................................36
31. Alar cells 2–3(–4)-stratose......................................................................................................32
31. Alar cells bistratose.................................................................................................................33
32. Epidermal cells on ventral side of costa differentiated.............................................................................................................................................*D. dispersum* Engelmark (in part)
32. Epidermal cells on ventral side of costa undifferentiated.............*D. crispifolium* Müll.Hal.
33. Laminal cells above strongly incrassate..*.............................................D. scottianum* Turner
33. Laminal cells thin-walled or slightly incrassate*..................................................................*34
34. Epidermal cells on ventral side of costa differentiated.......................*D. cheoi* E.B.Bartram
34. Epidermal cells on ventral side of costa not differentiated................................................35
35. Leaves falcate–secund.............................................................................*D. fuscescens* Turner
35. Leaves flexuous when dry and erect-spreading when moist...................................................................................................................*D. bardunovii* Tubanova & Ignatova
36. Transverse section of upper leaf keeled...........................*D. dispersum* Engelmark (in part)
36. Transverse section concave...................................................................................................37
37. Leaves ovate–lanceolate, with a short, narrowly acuminate apex…..*D. linzianum* C.Gao
37. Leaves narrowly lanceolate, with a long apex.....................................................................38
38. Leaves strongly curled when dry. Leaf margin near apex irregularly and coarsely denticulate to dentate.....................................................................*D. muehlenbeckii* Bruch & Schimp.
38. Leaves straight or weakly curled in their upper portions when dry. Leaf margin weakly denticulate to almost entirely smooth........................................................................................39
39. Plants more loosely tufted. Upper leaf laminal cells thin-walled to moderately incrassate; leaf base ovate or ovate–lanceolate....................................................................................40
39. Plants in dense, compact tufts. Upper leaf laminal cells strongly incrassate; leaf base lanceolate.......................................................................................................................................41
40. Plants small, leaves 4.5–5.7 × 0.73–0.83 mm................*D. schljakovii* Ignatova & Tubanova
40. Plants medium-sized, leaves 7–8 × 1.3–1.5 mm.............................*D. spadiceum* J.E.Zetterst.
41. Distal laminal cells short rectangular, quadrate, rounded........................................................................................................................*D. elongatum* Schleich. *ex* Schwägr.
41. Distal laminal cells elongated–rhomboidal...........................................*D. setifolium* Cardot
42. Capsules straight, peristome teeth smooth above, without being minutely and longitudinally point-striated below......................................................................*D. assamicum* Dixon
42. Capsules slightly curved. peristome teeth densely papillose above, minutely and longitudinally point-striated below........................................................*D. japonicum* Mitt. (in part)

## 3. Materials and Methods

### 3.1. Taxon Sampling

During the course of the present study, some specimens from the following Chinese herbaria were examined, including China Agricultural University (BAU), Central China Normal University (CCNU), East China Normal University (HSNU), Hangzhou Normal University (HTC), Institute of Botany, Chinese Academy of Sciences (PE), Kunming Institute of Botany, Chinese Academy of Sciences (KUN), Lushan Botanical Garden, Chinese Academy of Science (LBG), and Xinjiang University (XJU).

The inter-tribe relationships in *Dicranum* have been well resolved based on the combined analysis of five chloroplast loci (*rps*4-*trn*T, *trn*L-*trn*F, *trn*H-*psb*A, *rps*19-*rpl*2, and *rpo*B) and the nrITS1-5.8S-ITS2 region [4,7,11,12,14]. To determine the species phylogeny position of Chinese specimens, 29 specimens representing 19 species from China were newly sequenced for all six gene markers (see Supplementary Table S1). A total of 273 published accessions of *Dicranum*, spanning 36 species from previous studies, were also utilized for the analysis [4,5,6,7,8,11,12,14,38,75,76]. In addition, four accessions of *Holomitrium* Brid., two accessions of *Dicranoloma*, and one accession of *Fissidens* Hedw. were also selected as outgroups. A total of 309 accessions were included in the analysis. A list of taxa with collection localities, vouchers, herbarium acronyms, and GenBank accession numbers are listed in Supplementary Table S2.

### 3.2. Morphological Study

The plant photos were taken using a digital camera (Canon M6; Canon, Tokyo, Japan). Specimens were examined using a Zeiss stereo zoom scope (Stemi DV4; Zeiss, Oberkochen, Germany) and an Olympus microscope (Olympus BX43; Olympus, Tokyo, Japan), and microscopic images were captured by a digital camera (Olympus DP71; Olympus, Tokyo, Japan) attached to the microscope.

### 3.3. DNA Extraction, Sequencing, Assembly, and Annotation

The sample preparation, total DNA extraction, and chloroplast genome and nuclear DNA assembly followed protocols previously used in earlier studies [77]. The whole chloroplast genomes were annotated using Geneious v.11.1.5 [78] based on the *D. hengduanense* W.Z.Huang & R.L.Zhu as the reference plastome (accession number: OQ401775) and then adjusted manually. The assembled nuclear DNA was aligned with published ITS data using *D. scoparium* as a reference (accession number: KF423564) in Geneious v.11.1.5 [78], and then annotated and extracted.

### 3.4. Morphological Study

Six datasets were aligned using MAFFT v7.311 [79] and ambiguous alignment regions were trimmed using trimAl v1.2 [80]. Six individual alignments were concatenated in Geneious v.11.1.5 [78]. Absent bases were coded as missing.

Phylogenetic analyses were conducted using maximum likelihood (ML) and Bayesian inference (BI) methods in IQtree v2.0.6 [81] and MrBayes 3.2.6 [82], respectively. IQtree was performed with the best-fitting substitution model for each DNA region (HKY+F+G4 for ITS-partition, TPM2u+F+I+G4 for *rps*19-*rpl*2-partition, *rpo*B-partition, *rps*4-*trn*T-partition, and *trn*L-*trn*F intergenic spacers, and TPM3+F+G4 for t*rn*H-*psb*A-partition) selected by ModelFinder according to the Bayesian information criterion (BIC) [83,84], and the fast bootstrap option with 1000 replicates. For BI analyses, each DNA region was also assigned its own substitution model (HKY+G is the for best*-*fit model for ITS*-*partition; GTR+I+G for *rpo*B-partition and for *trn*L-*trn*F intergenic spacers; HKY+G for *rps*4-*trn*T-partition and for *rps*19-*rpl*2-partition; HKY+G for *trn*H-*psb*A*-*partition), as determined by the Akaike information criterion (AIC) [83,84]. Two independent analyses consisting of four Markov chain Monte Carlo (MCMC) chains were run for 5,000,000 generations, with one tree sampled for every 1000 generations. The posterior distribution of trees was summarized by a >50% majority-rule consensus tree after discarding the first 25% of samples as burn-in. Convergence was assessed by examining the likelihood plots in Tracer v.1.7 [85].

## 4. Conclusions

Four species of *Dicranum* are newly reported for China, including *D. bardunovii*, *D. dispersum*, *D. schljakovii*, and *D. spadiceum*. Two species of *Dicranum*, *D. brevifolium* and *D. viride*, are proposed for exclusion from the bryoflora of China. Additionally, *Dicranum psathyrum* is proposed as a synonym of *Dicranoloma fragile*. Currently, a total of 39 *Dicranum* species are known in China.

## Data Availability

Data are contained within the article and Supplementary Materials.

## Supplementary Materials

The following supporting information can be downloaded at: https://www.mdpi.com/article/10.3390/plants13131759/s1, Table S1: Sequences newly generated in the study, including taxon, isolate, locality, vouchers, herbarium code, and GenBank accession numbers; Table S2: Sequences downloaded from Genbank, including taxa, localities, vouchers, herbarium codes, and GenBank accession numbers (*rps*4*-trn*T, *trn*L*-trn*F, *trn*H*-psb*A, *rps*19*-rpl*2, *rpo*B, and ITS).
