# Supplementary material for: A Synopsis of Dicranum Hedw. (Dicranaceae, Bryophyta) in China, with Special References to Four Species Newly Reported and Re-Evaluation of Dicranum psathyrum Klazenga
Source: Plants (Basel). 2024 Jun 25;13(13):1759. doi: 10.3390/plants13131759 (PMC11243558; doi:10.3390/plants13131759)
Supplement: Supplementary file 1 [file plants-13-01759-s001.zip › Supplementary Table S1.pdf]

Supplementary Table S1. Sequences newly generated in the study, including taxon, isolate, locality, vouchers, herbarium code, and GenBank accession numbers. "—" means data missing.

Taxon	Isolate	Locality	Vouchers (herbarium code)	ITS	*rpo*B	*rps*4-*trn*T	*rps*19-*rpl*2	*trn*H-*psb*A	*trn*L-*trn*F
Dicranum bardunovii	H104	Yunnan, China	*R.L. Zhu et al. 20220830-30B* (HSNU)	PP680728	PP657896	PP657925	PP657954	PP657983	PP658012
Dicranum crispifolium	H215	Xizang, China	*X.M. Shao et al. 20200816SWZ010* (BAU)	PP680738	PP657906	PP657935	PP657964	PP657993	PP658022
Dicranum crispifolium	H73	Yunnan, China	*Q. Liu & Q. Zuo 1401* (HSNU)	PP680721	PP657889	PP657918	PP657947	PP657976	PP658005
Dicranum crispifolium	H108	Yunnan, China	*R.L. Zhu et al. 20220828-9* (HSNU)	PP680729	PP657897	PP657926	PP657955	PP657984	PP658013
Dicranum crispifolium	H190	Yunnan, China	*W.Z. Huang & S.H. Lu 20210905-51* (HSNU)	PP680733	PP657901	PP657930	PP657959	PP657988	PP658017
Dicranum dispersum	H90	Qinghai, China	*S.B. Zhang 20220709-41* (HSNU)	PP680727	PP657895	PP657924	PP657953	PP657982	PP658011
Dicranum flagellare	H85	Neimenggu, China	*R.L. Zhu et al. 20220802-4* (HSNU)	PP680724	PP657892	PP657921	PP657950	PP657979	PP658008
Dicranum fragilifolium	H83	Neimenggu, China	*R.L. Zhu et al.20220803-301* (HSNU)	PP680723	PP657891	PP657920	PP657949	PP657978	PP658007
Dicranum fragilifolium	H191	Xinjiang, China	*Mamtimin Sulayman 17145* (XJU)	PP680734	PP657902	PP657931	PP657960	PP657989	PP658018
Dicranum fragilifolium	H231	Xinjiang, China	*Mamtimin Sulayman 25902* (XJU)	PP680744	PP657912	PP657941	PP657970	PP657999	PP658028
Dicranum fuscescens	H226	Neimenggu, China	*R.L. Zhu et al. 20220803-305A* (HSNU)	PP680741	PP657909	PP657938	PP657967	PP657996	PP658025
Dicranum groenlandicum	H225	Heilongjiang, China	*R.L. Zhu et al. 20220804-83A* (HSNU)	PP680740	PP657908	PP657937	PP657966	PP657995	PP658024
Dicranum nipponense	H8	Yunnan, China	*R.L. Zhu et al. 20210603-17* (HSNU)	PP680719	PP657887	PP657916	PP657945	PP657974	PP658003
Dicranum polysetum	H82	Inner Mongolia, China	*R.L. Zhu et al. 20220803-307* (HSNU)	PP680722	PP657890	PP657919	PP657948	PP657977	PP658006
Dicranum psathyrum	H163	Jiangxi, China	*C84* (LBG, HSNU)	PP680730	PP657898	PP657927	PP657956	PP657985	PP658014
Dicranum schljakovii	H89	Neimenggu, China	*R.L. Zhu et al. 20220803-314* (HSNU)	PP680726	PP657894	PP657923	PP657952	PP657981	PP658010
Dicranum schljakovii	H229	Xinjiang, China	*Mamtimin Sulayman 9861* (XJU)	PP680742	PP657910	PP657939	PP657968	PP657997	PP658026
Dicranum spadiceum	H174	Qinghai, China	*L. Shu & W.Z. Huang 20220817-38* (HSNU)	PP680731	PP657899	PP657928	PP657957	PP657986	PP658015
Dicranum spadiceum	H187	Qinghai, China	*L. Shu & W.Z. Huang 20220817-39* (HSNU)	PP680732	PP657900	PP657929	PP657958	PP657987	PP658016
Dicranum spadiceum	H192	Xinjiang, China	*Mamtimin Sulayman 16887* (XJU)	PP680735	PP657903	PP657932	PP657961	PP657990	PP658019

Dicranum spadiceum	H193	Xinjiang, China	*Mamtimin Sulayman 26582* (XJU)	PP680736	PP657904	PP657933	PP657962	PP657991	PP658020
Dicranum spadiceum	H230	Xinjiang, China	*Horyat Abliz 450* (XJU)	PP680743	PP657911	PP657940	PP657969	PP657998	PP658027
Dicranum undulatum	H86	Heilongjiang, China	*R.L. Zhu et al 20220804-9* (HSNU)	PP680725	PP657893	PP657922	PP657951	PP657980	PP658009
Dicranoloma assimile	H197	Xizang, China	S.G. Tong 20221106-11 (CCNU, HSNU)	PP680737	PP657905	PP657934	PP657963	PP657992	PP658021
Dicranoloma braunii	H14	Yunnan, China	*R.L. Zhu et al. 20210602-16* (HSNU)	PP680720	PP657888	PP657917	PP657946	PP657975	PP658004
Dicranoloma cylindrothecium	H220	Zhejiang, China	*X.Y. Ma et al. 20220801-66* (HTC, HSNU)	PP680739	PP657907	PP657936	PP657965	PP657994	PP658023
Leucoloma molle	H244	Hainan, China	*J. Wang et al. 20210627-3* (HSNU)	—	PP657915	PP657944	PP657973	PP658002	PP658030
Leucophanes glaucum	H258	Laos	*S. He 43764* (KUN)	—	PP657913	PP657942	PP657971	PP658000	PP658029
Octoblepharum albidum	J294	Guangxi, China	*Zhu & Shen 20190824-32* (HSNU)	PP680745	PP657914	PP657943	PP657972	PP658001	PP658031
