# Supplementary material for: A Synopsis of Dicranum Hedw. (Dicranaceae, Bryophyta) in China, with Special References to Four Species Newly Reported and Re-Evaluation of Dicranum psathyrum Klazenga
Source: Plants (Basel). 2024 Jun 25;13(13):1759. doi: 10.3390/plants13131759 (PMC11243558; doi:10.3390/plants13131759)
Supplement: Supplementary file 1 [file plants-13-01759-s001.zip › Supplementary Table S2.pdf]

Supplementary Table S2. Sequences download from Genbank, including taxa, localities, vouchers, herbarium codes, and GenBank accession numbers (*rps*4-*trn*T, *trn*L-*trn*F, *trn*H-*psb*A, *rps*19-*rpl*2, *rpo*B, and ITS). "—" means data missing.

Taxon	Locality	vouchers (herbarium code)	rps4-trnT	trnL-trnF	trnH-psbA	rps19-rpl2	rpoB	ITS
Dicranoloma cylindrothecium		Y.J. Yoon s.n. (JNU)	—	KF423953	KF423490	—	KF423699	KF423621
Dicranoloma robustum		Frey, Schaumann 01-340 (F)	KF423822	KF423912	KF423445	—	—	DQ464190
Dicranum acutifolium I	Norway, Svalbard	Stech & Kruijer 10-102a (L)	KJ651006	KJ651057	KJ650789	KJ650961	KJ650913	KJ650855
D. acutifolium II	Norway, Svalbard	Stech & Kruijer 10-118 (L)	KJ651009	KJ651061	KJ650792	KJ650964	KJ650916	KJ650858
D. acutifolium III	Norway, Svalbard	Stech & Kruijer 08-031 (L)	KJ651013	KJ651069	KJ650799	KJ650968	KJ650923	KJ650865
D. acutifolium IV	Norway, Svalbard	Stech & Kruijer 08-033a (L)	KJ651014	KJ651070	KJ650800	KJ650969	KJ650924	KJ650866
D. acutifolium V	Norway, Svalbard	Stech & Kruijer 11-161 (L)	KJ651028	KJ651084	KJ650816	KJ650979	KJ650939	KJ650879
D. acutifolium VI	Finland, Kuusamo Prov.	Stech B970831.1 (L)	KJ651030	DQ462590	KJ650818	KJ650981	KJ650941	KJ650881
D. angustum I	Sweden, Jämtland	Hedenäs B193541 (S)	KJ651036	KJ651092	KJ650834	KJ650987	KJ650950	KJ650892
D. angustum II	Sweden, Jämtland	Hedenäs, Bisang & Persson B105001 (S)	—	KJ651090	KJ650832	KJ650986	KJ650948	KJ650890
D. angustum III	Sweden, Torne Lappmark	Norin B132922 (S)	KJ651037	KJ651093	KJ650835	KJ650988	KJ650951	KJ650893
D. angustum IV	Sweden, Torne Lappmark	Johansson B132926 (S)	KJ651038	KJ651094	KJ650836	KJ650989	KJ650952	KJ650894
D. angustum V	Sweden, Torne Lappmark	Norin B131031 (S)	—	KJ651091	KJ650833	—	KJ650949	KJ650891
D. angustum VI	Sweden, Dalarna	Hedenäs, Rönblom, Odelvik & Hamnede B139061 (S)	KM502592	KM502744	KM502622	KM502717	KM502697	KM502659
D. angustum VII	Sweden, Norrbotten	Westerberg B132876 (S)	KM502593	KM502745	KM502623	KM502718	KM502698	KM502660
D. angustum VIII	Sweden, Torne Lappmark	Johansson B132925 (S)	KM502594	KM502746	KM502624	KM502719	KM502699	KM502661
D. angustum IX	Sweden, Jämtland	Hedenäs B107583 (S)	—	KM502747	KM502625	KM502720	KM502700	KM502662
D. baicalense I	Russia, Republic of Buryatia	Tubanova Kyakh-6/1042 (UUH)	OQ060652	OP948648	—	—	—	OP939925
D. baicalense II	Russia, Republic of Buryatia	Tubanova O1517/01 (UUH)	—	OP948649	—	—	—	OP939927
D. baicalense III	Russia, Zabaikalskiy Territory	Afonina 7912b (LE, UUH)	—	OP948650	—	—	—	OP939926

D. baicalense IV	Russia, Amur Region	Bezgodov 261 (PPU, UUH)	OQ060653	OP948651	—	—	—	OP939929
D. baicalense V	Russia, Primorskiy Territory	Tubanova Pr1508/02 (UUH)	—	OP948652	—	—	—	OP939928
Dicranum baicalense VI	China, Inner Mongolia	*R.-L. Zhu et al. 20220802-39* (HSNU)	PP101798	PP101804	PP101810	PP101816	PP101822	PP096848
D. bonjeanii I	Switzerland, Geneva	Lang, Price & Naciri 20070523.21 (G)	GQ428105	GQ428059	GQ428014	GQ427976	GQ427936	KF423636
D. bonjeanii II	France, Savoie	Hovenkamp 10/43 (L)	KF423849	KF423937	KF423474	—	KF423683	KF423581
D. bonjeanii III	France, Savoie	Hovenkamp 10/47 (L)	KF423850	KF423938	KF423475	KF423776	KF423684	KF423582
D. bonjeanii IV	France, Savoie	Hovenkamp 10/44 (L)	KF423851	KF423939	KF423476	—	KF423685	KF423583
D. bonjeanii V	Netherlands, South Holland	Lang 20091126.2 (L)	KF423871	KF423965	KF423502	KF423798	KF423710	KF423608
D. bonjeanii VI	Sweden, Torne Lappmark	Norin B132878 (S)	KM502595	KM502748	KM502626	KM502721	KM502701	KM502663
D. bonjeanii VII	China, Yunnan	R.-L. Zhu et al. 20220830-33 (HSNU)	PP101799	PP101805	PP101811	PP101817	PP101823	PP096849
D. brevifolium I	Russia, Karachaevo-Cherkessia Prov.	Egorov isolate B2 (MW)	KJ796611	KJ796587	KJ796520	—	KJ796563	HQ830342
D. brevifolium II	Russia, Tuva Prov.	Otnyukova isolate B4 (KRF)	KJ796612	KJ796588	KJ796521	KJ796629	KJ796564	HQ830341
D. brevifolium III	Russia, North Ossetia	Korotko isolate B3 (MW)	—	KJ796589	KJ796522	KJ796630	KJ796565	HQ830343
D. brevifolium IV	Switzerland, Wallis	Hedenäs B98890 (S)	KJ651039	KJ651095	KJ650837	KJ650990	KJ650953	KJ650895
D. brevifolium V	Sweden, Hälsingland	Hedenäs B175744 (S)	KJ651040	KJ651096	KJ650838	KJ650991	KJ650954	KJ650896
D. cf scoparium I	U.S.A., Pennsylvania, Cambria Co.	Davis 270 (MO)	GU068416	GU068500	GU068471	GU068387	GU068443	KF423567
D. cf scoparium II	U.S.A., Kentucky, Greenup Co.	Risk, Richardson &Newland 14463 (MO)	GU068413	GU068497	GU068468	GU068384	GU068440	KF423571
D. cf scoparium III	U.S.A., Maine, Knox Co.	Allen 28074 (MO)	GU068414	GU068498	GU068469	GU068385	GU068441	KF423572
D. cf scoparium IV	U.S.A., Missouri, Jefferson Co.	Holmberg 1578 (MO)	GU068415	GU068499	GU068470	GU068386	GU068442	KF423573

D. cf scoparium V	Canada, Nova scotia, Digby Co.	Schofield & Schofield 95348 (UBC)	—	KF424001	KF423540	—	—	KF423647
D. cf. *lorifolium* I	Russia, Primorsky Prov.	Lang 20100910.8 (L)	KF423909	KF424010	KF423549	— —	KF423750	KF423655
D. cf. *lorifolium* II	Russia, Primorsky Prov.	Lang 20100910.10 (L)	KF423910	KF424011	KF423550	— —	KF423751	KF423656
D. cf. *lorifolium* III	Russia, Primorsky Prov.	Lang 20100909.9 (L)	KF423911	KF424012	KF423551	KF423821	— —	— —
D. cf. *lorifolium* IV	Russia, Primorsky Prov.	Lang 20100905.10 (L)	KF423907	KF424008	KF423547	— —	— —	KF423653
D. cf. *lorifolium* V	Russia, Primorsky Prov.	Lang and Cherdantseva 20100908.14 (L)	KF423906	KF424007	KF423546	— —	KF423748	KF423652
D. cf. *lorifolium* VI	Russia, Primorsky Prov.	Lang 20100906.17 (L)	KF423904	KF424005	KF423544	— —	KF423746	KF423650
D. cf. *lorifolium* VII	Russia, Primorsky Prov.	Lang 20100906.6 (L)	KF423903	KF424004	KF423543	— —	— —	KF423649
D. cf. *lorifolium* VIII	Russia, Primorsky Prov.	Lang 20100906.11 (L)	KF423908	KF424009	KF423548	— —	KF423749	KF423654
D. cf. *lorifolium* IX	Russia, Primorsky Prov.	Lang 20100906.8 (L)	KF423905	KF424006	KF423545	— —	KF423747	KF423651
D. crassifolium I	Portugal, Trás-os-Montes e Alto Douro	Sérgio FRID 13g (LISU)	KM502599	KM502752	KM502630	—	—	KM502667
D. crassifolium II	Portugal, Beira Litora	Sérgio 13796 (LISU)	—	KM502753	KM502631	—	—	KM502668
D. crassifolium III	Portugal, Beira Alta	Sérgio 14679 (LISU)	KM502600	KM502754	KM502632	KM502725	—	KM502669
D. crassifolium IV	Portugal, Douro Litoral	Garcia 205276 (LISU)	—	KM502755	KM502633	KM502726	—	KM502670
D. crassifolium V	Portugal, Trás-os-Montes e Alto Douro	Sérgio, Carvalho, Garcia & Louro 212140 (LISU)	—	KM502756	KM502634	—	—	KM502671
D. dispersum I	Germany, Baden-Württemberg	Sauer MS95022 (S)	KJ651041	KJ651097	KJ650839	KJ650992	KJ650955	KJ650897
D. dispersum II	Russia, Zabaikalskiy Territory	Afonina 8912 (LE)	—	—	—	—	—	KT580734
D. dispersum III	Russia, Primorskiy Territory	Ignatov 08-317 (MHA)	—	KT580692	—	—	—	KT580746
D. dispersum IV	Russia, Dagestan	Ignatov & Ignatova 09-189 (MHA)	—	KT580686	—	—	—	KT580740
D. dispersum V	USA, Alaska	Breen T001-3B (LE)	—	KT580685	—	—	—	KT580739
D. dispersum VI	Russia, Buryatia	Tubanova Кях-6/10 (UUH)	—	KT580684	—	—	—	KT580738
D. dispersum VII	Russia, Irkutsk Province	Dudareva 04-35 (IRK)	—	KT580683	—	—	—	KT580737

D. dispersum VIII	Russia, Buryatia	Krivobokov оп.113 (UUH)	—	KT580682	—	—	—	KT580736
D. dispersum IX	Russia, Ingushetia	Bersanova s.n. (MHA)	—	KT580681	—	—	—	KT580735
D. drummondii	Finland, Kuusamo Prov.	Stech B970827.4 (L)	KJ796609	DQ462589	KJ796518	—	KJ796561	KJ796538
D. elongatum I	Norway, Svalbard	Stech & Kruijer 11-213 (L)	KJ651027	KJ651083	KJ650815	KJ650978	KJ650938	KJ650878
D. elongatum II	Finland, Kuusamo Prov.	Stech B970831.3 (L)	KJ651031	DQ462592	KJ650819	KJ650982	KJ650942	KJ650882
D. elongatum III	Norway, Svalbard	Stech & Kruijer 08-250 (L)	KJ651008	KJ651059	KJ650791	KJ650963	KJ650915	KJ650857
D. elongatum IV	Norway, Svalbard	Stech & Kruijer 10-202 (L)	—	KJ651062	KJ650793	—	KJ650917	KJ650859
D. flagellare I	Netherlands, Utrecht	Wondergem 1300 (L)	KM502601	KM502757	KM502635	KM502727	—	KM502672
D. flagellare II	Netherlands, Limburg	Bijlsma 12053 (L)	KM502602	KM502758	KM502636	KM502728	—	KM502673
D. flagellare III	Netherlands, Gelderland	Bijlsma 13104 (L)	KM502603	KM502759	KM502637	KM502729	—	KM502674
D. flexicaule IV	Russia, Tuva Prov.	Molokova isolate PA3 (KRF)	KJ796606	KJ796581	KJ796513	—	KJ796555	HQ830328
D. flexicaule V	Russia, Krasnoyarsk Territory	Otnyukova isolate PA1 (KRF)	—	KJ796582	KJ796514	—	KJ796556	HQ830330
D. flexicaule VI	Russia, Zabaikalsky Territory	Dudareva isolate FL1 (IRK)	KJ796607	KJ796583	KJ796515	—	KJ796557	HQ830331
D. flexicaule VII	Russia, Primorsky Territory	Ignatov, Ignatova & Cherdanzeva 06–2637 (MHA)	—	KJ796584	—	—	KJ796558	HQ830332
D. flexicaule VIII	U.S.A., Alaska, Toolik Lake	Morgado & Geml Bry 280712 001 (L)	KJ651000	KJ651051	KJ650824	—	KJ650907	KJ650849
D. flexicaule IX	U.S.A., Alaska, Toolik Lake	Morgado & Geml Bry 280712 002 (L)	KJ651001	KJ651052	KJ650825	—	KJ650908	KJ650850
D. flexicaule X	U.S.A., Alaska, Toolik Lake	Morgado & Geml Bry 280712 003 (L)	KJ651002	KJ651053	KJ650826	—	KJ650909	KJ650851
D. flexicaule XI	U.S.A., Alaska, Toolik Lake	Morgado & Geml Bry 280712 004 (L)	KJ651003	KJ651054	KJ650827	—	KJ650910	KJ650852
D. flexicaule XII	U.S.A., Alaska, Toolik Lake	Morgado & Geml Bry 280712 005 (L)	KJ651004	KJ651055	KJ650828	—	KJ650911	KJ650853
D. flexicaule XIII	U.S.A., Alaska, Toolik Lake	Morgado & Geml Bry 280712 006 (L)	KJ651005	KJ651056	KJ650829	—	KJ650912	KJ650854

D. flexicaule XIV	Norway, Troms	Hassel B–6135 (TRH)	—	KF423969	KF423506	—	KF423714	KF423612
D. flexicaule XV	Finland, Kuusamo Prov.	Stech B970827.5 (L)	KJ651032	—	KJ650820	—	KJ650943	KJ650883
D. fragilifolium I	Russia, Vologda Prov.	Ignatov & Ignatova s.n. (MW)	KM502604	KM502761	KM502639	—	KM502706	FJ952596
D. fragilifolium II	Russia, Arkhangelsk Prov.	Churakova s.n. (MW)	—	KM502760	KM502638	—	KM502705	FJ952597
D. fragilifolium III	Finland, Kuusamo Prov.	Stech B970828.8 (L)	—	KM502762	—	—	—	KM502675
D. fragilifolium IV	Finland, Kuusamo Prov.	Stech B970827.1 (L)	KF423837	AF135069/ AF136077	KF423462	KF423766	KF423673	AF140700
D. fuscescens I	Russia, Perm Prov.	Bezgodov & Shkaraba isolate FU1 (MHA)	—	KJ796578	KJ796510	—	KJ796552	HQ830334
D. fuscescens II	Russia, Primorsky Territory	Ignatov, Ignatova & Cherdanzeva 06–2588 (MHA)	KJ796605	KJ796579	KJ796511	—	KJ796553	HQ830337
D. fuscescens III	Russia, Sakhalin	Ignatov & Teleganova 44726 (MHA)	—	KJ796580	KJ796512	—	KJ796554	HQ830335
D. fuscescens IV	Netherlands, Gelderland	Wondergem 1134 (L)	KJ651042	KJ651098	KJ650840	KJ650993	—	KJ650898
D. fuscescens V	Finland, Karelia Prov.	Stech B970824.3 (L)	KF423896	—	KF423534	KF423819	KF423742	KF423642
D. groenlandicum I	Sweden, Torne Lappmark	Hedenäs B74363 (S)	—	KJ651089	KJ650830	KJ650984	KJ650946	KJ650888
D. groenlandicum II	Sweden, Jämtland	Hedenäs B74365 (S)	—	KM502763	KM502640	—	KM502707	KM502676
D. howellii I	U.S.A., Alaska	Talbot TAN 1C-17 (UBC)	— —	KF423914	KF423447	— —	KF423658	— —
D. howellii II	U.S.A., California	Shevock 19290 (MO)	KF423841	KF423929	KF423466	KF423769	KF423676	KF423570
D. howellii III	U.S.A., California	Allen 24114 (MO)	KF423840	KF423928	KF423465	— —	— —	KF423569
D. howellii IV	U.S.A., Oregon	Allen 28834 (MO)	KF423843	KF423931	KF423468	KF423770	KF423677	KF423575
D. howellii V	U.S.A., Washington	Schofield. and Harpel 120527 (UBC)	KF423891	KF423991	KF423529	KF423817	KF423737	KF423637
D. japonicum I	South Koera	Yoon s.n. (JUN)	KF423840	KF423928	KF423465	— —	— —	KF423569
D. japonicum II	South Koera	Yoon s.n. (JUN)	KF423843	KF423931	KF423468	KF423770	KF423677	KF423575
D. japonicum III	South Koera	Yoon s.n. (JUN)	KF423891	KF423991	KF423529	KF423817	KF423737	KF423637
D. japonicum IV	South Koera	Yoon s.n. (JUN)	KF423864	KF423954	,KF423491	— —	KF423700	KF423598
D. japonicum V	South Koera	Yoon s.n. (JUN)	KF423865	KF423955	KF423492	KF423790	KF423701	KF423599
D. laevidens I	Norway, Svalbard	Stech & Kruijer 10-216 (L)	KJ651010	KJ651063	KJ650794	KJ650965	KJ650918	KJ650860

D. laevidens II	Norway, Svalbard	Stech & Kruijer 10-002 (L)	KJ651011	KJ651064	KJ650795	—	KJ650919	KJ650861
D. laevidens III	Norway, Svalbard	Stech & Kruijer 10-006a (L)	—	KJ651065	KJ650796	—	KJ650920	KJ650862
D. laevidens IV	Norway, Svalbard	Stech & Kruijer 09-71 (L)	—	KJ651067	KJ650808	—	—	KJ650874
D. laevidens V	Norway, Svalbard	Stech & Kruijer 09-021 (L)	KJ651015	KJ651071	KJ650801	—	KJ650925	KJ650867
D. laevidens VI	Norway, Svalbard	Stech & Kruijer 09-022 (L)	KJ651016	KJ651072	KJ650802	—	KJ650926	KJ650868
D. laevidens VII	Norway, Svalbard	Stech & Kruijer 11-0431 (L)	KJ651025	KJ651081	KJ650813	KJ650976	KJ650936	KJ650876
D. laevidens VIII	Sweden, Jämtland	Hedenäs, Bisang & Persson B105000 (S)	KJ651035	—	KJ650831	KJ650985	KJ650947	KJ650889
D. laevidens IX	Sweden, Lule Lappmark	Westerberg B131027 (S)	—	KM502764	KM502641	KM502730	KM502708	KM502677
D. laevidens X	Sweden, Dalarna	Hedenäs B85000 (S)	—	KM502765	KM502642	—	—	KM502678
D. laevidens XI	Sweden, Jämtland	Hedenäs B74327 (S)	KM502605	KM502766	KM502643	KM502731	KM502709	KM502679
D. leioneuron I	Sweden, Medelpad	Hedenäs B116708 (S)	KJ651048	KJ651104	KJ650846	—	KJ650958	KJ650904
D. leioneuron II	Sweden, Hälsingland	Laegaard, Gustafsson, Poulsen, Brunbjerg 23200L (S)	KJ651049	KJ651105	KJ650847	KJ650998	KJ650959	KJ650905
D. leioneuron III	Sweden, Dalsland	Hedenäs & Persson B135011 (S)	KJ651050	KJ651106	KJ650848	KJ650999	KJ650960	KJ650906
D. majus I	U.S.A., Alaska, Attu Island.	Talbot ATT102-30 (MO)	GU068421	GU068504	GU068476	GU068392	GU068447	KF423574
D. majus II	Finland, Kuusamo Prov.	Stech B970828.9 (L)	—	KJ651087	KJ650822	—	—	KJ650886
D. majus III	Norway, Svalbard	Stech & Kruijer 10-029 (L)	KF423823	KF423913	KF423446	—	KF423657	KF423552
D. majus IV	U.S.A., Alaska, Sea Parrot Island	Talbot & Schofield ADA42-29 (UBC)	KF423824	KF423915	KF423448	KF423752	KF423659	KF423553
D. majus V	U.S.A., Alaska, Selawik Nat. Wildlife Refuge	Talbot &. Solomeschch 05-41-18 (UBC)	KF423825	KF423916	KF423449	KF423753	KF423660	KF423554
D. majus VI	U.S.A., Alaska, Attu Island	Schofield &Talbot 120253 (UBC)	—	KF423922	KF423455	KF423759	KF423666	KF423560
D. majus VII	Russia, Primorsky Prov.	Lang 20100906.2 (L)	KF423879	KF423977	KF423515	KF423808	KF423723	KF423620
D. majus VIII	Finland, Kuusamo Prov.	Stech B970829.4 (L)	KF423836	AF135068/ AF136076	KF423461	KF423765	KF423672	AF144114
D. montanum I	Germany,	Stech B890721.5 (L)	KF423878	AF129589/	KF423512	KF423805	KF423720	AF144115

	Nordrhein-Westfalen			AF129562				
D. montanum II	Netherlands, North Holland	Wondergem 1302 (L)	KM502606	KM502767	KM502644	KM502732	—	KM502680
D. montanum III	Netherlands, Gelderland	Zwarts 2033 (L)	KM502607	KM502768	KM502645	KM502733	—	KM502681
D. montanum IV	Netherlands, North Brabant	Smulders 10139 (L)	KM502608	KM502769	KM502646	KM502734	—	KM502682
D. nipponense I	Russia, Primorsky Prov.	Lang 20100909.1 (L)	KF423882	KF423981	KF423519	KF423810	KF423727	KF423624
D. nipponense II	Russia, Primorsky Prov.	Lang 20100909.1 (L)	KF423883	KF423982	KF423520	KF423811	KF423728	KF423625
D. polysetum I	Germany, Mecklenburg-Vorpommern	Stech B9705181.1 (L)	KF423838	AF129587	EU163523	KF423767	KF423674	AF144113
D. polysetum II	Netherlands, Utrecht	Wondergem 1355 (L)	KM502609	KM502770	KM502647	KM502735	—	KM502683
D. polysetum III	Netherlands, Gelderland	Zwarts 2121 (L)	KM502610	KM502771	KM502648	KM502736	—	KM502684
D. polysetum IV	Netherlands, Overijssel	Aptroot 69434 (L)	KM502611	KM502772	—	KM502737	—	KM502685
D. scoparium I	Switzerland, Geneva	Lang 20080907.1 (G)	GQ428082	GQ428036	GQ427991	GQ427953	GQ427914	KF423564
D. scoparium II	Switzerland, Geneva	Lang 20080907.6 (G)	GU068393	GU068477	GU068448	GU068364	GU068422	KF423565
D. scoparium III	Canada, Newfoundland, Avalon Peninsula.	Allen 28704 (MO)	GU068418	GU068502	GU068473	GU068389	GU068445	KF423568
D. scoparium IV	Switzerland, Vaud	Lang & Price 20080701.1 (G)	GQ428088	GQ428041	GQ427997	GQ427960	GQ427919	KF423584
D. scoparium V	Switzerland, Geneva	Lang & Price 20070719.31 (G)	GU068406	GU068490	GU068461	GU068377	GU068434	KF423634
D. scoparium VI	Switzerland, Geneva	Lang & Price 20070719.35 (G)	GQ428101	GQ428056	GQ428011	GQ427973	GQ427933	KF423635
D. scoparium VII	Finland,	Stech s.n. (L)	—	KJ651086	KJ650821	—	KJ650945	KJ650884
D. scoparium VIII	Spain, Ibias	Fdez. Ordóñez 269 (FCO-Briof)	KF423826	KF423917	KF423450	KF423754	KF423661	KF423555
D. scoparium IX	Spain, Cangas del Narcea	Fdez. Ordóñez 1077 (FCO-Briof)	KF423827	KF423918	KF423451	KF423755	KF423662	KF423556
D. scoparium X	Spain, Cangas de Onís	del Collado 688119 (FCO-Briof)	KF423828	KF423919	KF423452	KF423756	KF423663	KF423557
D. scoparium XI	Spain, Parque Nacional Picos de Europa	del Collado 399213 (FCO-Briof)	KF423829	KF423920	KF423453	KF423757	KF423664	KF423558
D. scoparium XII	Spain, Cabrales; Picos de Europa	del Collado 673509 (FCO-Briof)	KF423830	KF423921	KF423454	KF423758	KF423665	KF423559
D. scoparium XIII	Canada, British Columbia,	Schofield & Klinkenberg 119252	KF423898	KF423997	KF423536	—	—	KF423644

	Lulu Island	(UBC)						
D. scoparium XIV	Canada, British Columbia, Lac le Jeune Road	Schofield & Williams 117252A (UBC)	KF423899	KF423998	KF423537	—	—	KF423645
D. scoparium XV	Canada, Ontario, Bruce Co.	Buck 54100 (NY)	KF423831	KF423923	KF423456	KF423760	KF423667	KF423561
D. scoparium XVI	Bulgaria, Sofia Prov.	Papp 10/101/1 (L)	KF423832	KF423924	KF423457	KF423761	KF423668	KF423562
D. scoparium XVII	Russia, Karachaevo-Cherkessian Rep.	Ignatov & Ignatova B113001 (S)	KF423833	KF423925	KF423458	KF423762	KF423669	KF423563
D. scoparium XVIII	France, Haute–Corse	Sotiaux & Sotiaux 462 (S)	KF423834	KF423926	KF423459	KF423763	KF423670	KF423566
D. scoparium XIX	Canada, Nova Scotia, Richmond Co.	King & Garvey B657 (MO)	KF423835	KF423927	KF423460	KF423764	KF423671	—
D. scoparium XX	Canada, Ontario, Thunder bay district	Allen 9479 (L)	—	KF423999	KF423538	—	—	KF423646
D. scoparium XXI	Portugal, Madeira	Stech 04–217 (L)	KF423844	KF423932	KF423469	KF423771	KF423678	KF423576
D. scoparium XXII	Germany, Northrhine-Westphalia	Stech 09–317 (L)	KF423845	KF423933	KF423470	KF423772	KF423679	KF423577
D. scoparium XXIII	Germany, Northrhine-Westphalia	Stech 09–318 (L)	KF423846	KF423934	KF423471	KF423773	KF423680	KF423578
D. scoparium XXIV	Germany, Rhineland–Palatinate	Stech 10–009 (L)	KF423847	KF423935	KF423472	KF423774	KF423681	KF423579
D. scoparium XXV	France, Alpes–Maritimes	Martinez s.n. (L)	KF423848	KF423936	KF423473	KF423775	KF423682	KF423580
D. scoparium XXVI	Canada, Ontario, Gloucester	Ireland, Dugal & Ley 23775 (CANM)	—	KF423940	KF423477	KF423777	KF423686	KF423585
D. scoparium XXVII	Greece, Central Macedonia	Papp 10/77/6 (L)	KF423852	KF423941	KF423478	KF423778	KF423687	KF423586
D. scoparium XXVIII	Hungary, Northern Hungary	Schofield 104660 (UBC)	KF423853	KF423942	KF423479	KF423779	KF423688	KF423587
D. scoparium XXIX	Iceland, Norðurland eystra	Elmarsdóttir 42630 (ICEL)	KF423854	KF423943	KF423480	KF423780	KF423689	KF423588

D. scoparium XXX	Iceland, Norðurland vestra	Egilsson 44119 (ICEL)	KF423862	KF423951	KF423488	KF423788	KF423697	KF423596
D. scoparium XXXI	Iceland, Norðurland eystra	Egilsson 44218 (ICEL)	KF423855	KF423944	KF423481	KF423781	KF423690	KF423589
D. scoparium XXXII	Iceland, Norðurland eystra	Elmarsdóttir 44446 (ICEL)	KF423856	KF423945	KF423482	KF423782	KF423691	KF423590
D. scoparium XXXIII	Iceland, Norðurland eystra	Þórisson 43747 (ICEL)	KF423857	KF423946	KF423483	KF423783	KF423692	KF423591
D. scoparium XXXIV	NL, Norðurland vestra	Lang 20091203.3 (L)	KF423863	KF423952	KF423489	KF423789	KF423698	KF423597
D. scoparium XXXV	South Korea, Jeju–do Hallasan	Yoon s.n. (JNU)	KF423866	KF423956	KF423493	KF423791	KF423702	KF423600
D. scoparium XXXVI	Canada, Ontario, Lennox & Addinton Co.	Ley & al. 1222 (CANM)	—	KF423959	KF423496	KF423792	KF423705	KF423603
D. scoparium XXXVII	Macedonia, Pelagonia region	Papp 10/87/2 (L)	KF423869	KF423960	KF423497	KF423793	KF423706	KF423604
D. scoparium XXXVIII	Portugal, Madeira	Hedenäs B4566 (S)	—	KF423961	KF423498	KF423794	KF423707	KF423605
D. scoparium XXXIX	Portugal, Madeira	Hedenäs & Bisang B22461 (S)	—	KF423962	KF423499	KF423795	—	KF423606
D. scoparium XL	Portugal, Madeira	Stech 04–576 (L)	KF423870	KF423963	KF423500	KF423796	KF423708	KF423607
D. scoparium XLI	Netherlands, South Holland	van den Vaart s.n. (L)	KF423872	KF423966	KF423503	KF423799	KF423711	KF423609
D. scoparium XLII	Norway, Herøy	Prestø B-7239 (TRH)	KF423873	KF423967	KF423504	KF423800	KF423712	KF423610
D. scoparium XLIII	Norway, Gloppen	Hassel B-6584 (TRH)	—	KF423968	KF423505	—	KF423713	KF423611
D. scoparium XLIV	Norway, Gjemnes	Prestø B-7017 (TRH)	—	KF423970	KF423507	—	KF423715	KF423613
D. scoparium XLV	Norway, Frei	Prestø B-7605 (TRH)	KF423874	KF423971	KF423508	KF423801	KF423716	KF423614
D. scoparium XLVI	Norway, Buskerud	Hanssen 753983 (O)	KF423875	KF423972	KF423509	KF423802	KF423717	KF423615
D. scoparium XLVII	Norway, Østfold	Engan GE-20 (O)	KF423876	KF423973	KF423510	KF423803	KF423718	KF423616
D. scoparium XLVIII	Norway, Buskerud	Hanssen 5399 (O)	KF423877	KF423974	KF423511	KF423804	KF423719	KF423617
D. scoparium XLIX	Canada, Ontario, Parry Sound	Ireland 23915 (CANM)	—	KF423975	KF423513	KF423806	KF423721	KF423618

D. scoparium L	Portugal, Coimbra	Hedenäs B44512 (S)	—	KF423976	KF423514	KF423807	KF423722	KF423619
D. scoparium LI	Sweden, Gästrikland	Odelvik B163166 (S)	KF423886	KF423986	KF423524	KF423812	KF423732	KF423629
D. scoparium LII	Sweden, Jämtland	Hedenäs B164630 (S)	KF423887	KF423987	KF423525	KF423813	KF423733	KF423630
D. scoparium LIII	Netherlands, North Holland	Lang 20100314.1 (L)	KF423888	KF423988	KF423526	KF423814	KF423734	KF423631
D. scoparium LIV	Germany, Schleswig–Holstein	Stech B960719.1 (L)	KF423839	AF129588/ AF129561	KF423464	KF423768	KF423675	AF140699
D. scoparium LV	U.S.A., Colorado, San Juan Co.	Weber, Wittmann, Andrus & Cooper B–111031 (MO)	—	KM502773	KM502649	—	—	KM502686
D. scoparium LVI	U.S.A., Oregon, Umatilla National Forest	Schofield, Harpel & Forest Service Personnel 116776 (UBC)	KM502612	KM502774	KM502650	—	KM502710	KM502687
D. scoparium LVII	Russia, Irkutsk Prov.	van Melick 214110 (L)	KM502613	KM502775	KM502651	—	KM502711	KM502688
D. scoparium LVIII	U.S.A., South Dakota, Pennington Co.	Churchill & Churchill 19597 (UBC)	KM502614	KM502776	KM502652	—	—	KM502689
D. scoparium LIX	Netherlands, North Holland	Lang 20100314.2 (L)	KF423889	KF423989	KF423527	KF423815	KF423735	KF423632
D. scoparium LX	Netherlands, North Holland	Lang 20100314.3 (L)	KF423890	KF423990	KF423528	KF423816	KF423736	KF423633
D. scoparium LXI	China, Xinjiang	Sulayman 35804 *(XJU)*	PP101800	PP101806	PP101812	PP101818	PP101824	PP096850
D. scoparium LXII	China, Xinjiang	Sulayman 17371 *(XJU)*	PP101801	PP101807	PP101813	PP101819	PP101825	PP096851
D. scottianum I	Portugal, Azores	Waltje AZ-0184 (Herb. H. Waltje)	KJ651047	KJ651103	KJ650845	—	KJ650957	KJ650903
D. scottianum II	Portugal, Azores	Waltje AZ-0102 (Herb. H. Waltje)	KJ651046	KJ651102	KJ650844	—	KJ650956	KJ650902
D. scottianum III	Spain, Canaries Islands	Stech 04-405 (L)	KM502596	KM502749	KM502627	KM502722	KM502702	KM502664
D. scottianum IV	Spain, Canaries Islands	Stech 04-547 (L)	KM502597	KM502750	KM502628	KM502723	KM502703	KM502665
D. scottianum V	Spain, Canaries Islands	Stech 07-113 (L)	KM502598	KM502751	KM502629	KM502724	KM502704	KM502666
D. septentrionale I	Russia, Kamchatka	Neshataeva 986 (LE)	KJ796608	KJ796585	KJ796516	KJ796627	KJ796559	HQ830338
D. septentrionale II	Russia, Arkhangelsk Prov.	Churakova 864 (MW)	—	KJ796586	KJ796517	—	KJ796560	HQ830339
D. septentrionale III	Sweden, Uppland	Hedenäs B74004 (S)	KJ796613	KJ796590	KJ796523	KJ796631	KJ796566	KJ796540
D. septentrionale IV	Sweden, Härjedalen	Hedenäs B122921 (S)	KJ796614	KJ796591	KJ796524	KJ796632	KJ796567	KJ796541

D. septentrionale V	Sweden, Gotland	Hedenäs B183369 (S)	KJ796615	KJ796592	KJ796525	KJ796633	—	KJ796542
D. septentrionale VI	Sweden, Södermanland	Hedenäs B193369 (S)	KJ796616	KJ796593	KJ796526	KJ796634	KJ796568	KJ796543
D. septentrionale VII	Sweden, Torne Lappmark	Hallingbäck 46166 (S)	KJ796617	KJ796594	KJ796527	KJ796635	KJ796569	KJ796544
D. septentrionale VIII	Sweden, Gotland	Hedenäs & Bisang B84948 (S)	KJ796618	KJ796595	KJ796528	KJ796636	KJ796570	KJ796545
D. septentrionale IX	Austria, Tirol	Stech B960801.2 (L)	KJ796610	DQ462591	KJ796519	KJ796628	KJ796562	KJ796539
D. shennongjiaense I	China, Hubei	He & Yang 45 (CCNU, HSNU)	PP101802	PP101808	PP101814	PP101820	PP101826	PP098990
D. shennongjiaense II	China, Hubei	Long 78 (CCNU, HSNU)	PP101803	PP101809	PP101815	PP101821	PP101827	PP098991
D. spadiceum I	Norway, Svalbard	Stech & Kruijer 08-203 (L)	KJ651007	KJ651058	KJ650790	KJ650962	KJ650914	KJ650856
D. spadiceum II	Norway, Svalbard	Stech & Kruijer 10-297 (L)	KJ651012	KJ651066	KJ650797	KJ650966	KJ650921	KJ650863
D. spadiceum III	Norway, Svalbard	Stech & Kruijer 08-025 (L)	—	KJ651068	KJ650798	KJ650967	KJ650922	KJ650864
D. spadiceum IV	Norway, Svalbard	Stech & Kruijer 10-046 (L)	KJ651017	KJ651073	KJ650803	KJ650970	KJ650927	KJ650869
D. spadiceum V	Norway, Svalbard	Stech & Kruijer 11-229 (L)	KJ651018	KJ651074	KJ650804	KJ650971	KJ650928	KJ650870
D. spadiceum VI	Norway, Svalbard	Stech & Kruijer 11-237 (L)	KJ651019	KJ651075	KJ650805	KJ650972	KJ650929	KJ650871
D. spadiceum VII	Norway, Svalbard	Stech & Kruijer 11-171 (L)	KJ651020	KJ651077	KJ650806	KJ650973	KJ650930	KJ650872
D. spadiceum VIII	Norway, Svalbard	Stech & Kruijer 11-153 (L)	KJ651026	KJ651082	KJ650814	KJ650977	KJ650937	KJ650877
D. spadiceum IX	Norway, Svalbard	Stech & Kruijer 11-165 (L)	KJ651029	KJ651085	KJ650817	KJ650980	KJ650940	KJ650880
D. spadiceum X	Norway, Svalbard	Stech & Kruijer 11-167b (L)	KJ651021	KJ651079	KJ650807	KJ650974	KJ650931	KJ650873
D. spadiceum XI	Norway, Svalbard	Stech & Kruijer 11-167c (L)	KJ651024	KJ651080	KJ650812	—	KJ650935	KJ650875
D. spadiceum XII	Iceland, Norðurland vestra	Egilsson 44012 (ICEL)	KF423858	KF423947	KF423484	KF423784	KF423693	KF423592
D. spadiceum XIII	Iceland, Norðurland eystra	Egilsson 43998 (ICEL)	KF423859	KF423948	KF423485	KF423785	KF423694	KF423593
D. spadiceum XIV	Iceland, Norðurland eystra	Egilsson 41447 (ICEL)	KF423860	KF423949	KF423486	KF423786	KF423695	KF423594
D. spadiceum XV	Iceland, Norðurland vestra	Egilsson 43804 (ICEL)	KF423861	KF423950	KF423487	KF423787	KF423696	KF423595
D. spadiceum XVI	Russia, Caucasus	Ukrainskaya, #14644 (LE)	—	KT580713	—	—	—	KT580766
D. spadiceum XVII	Russia, Irkutsk Province	Kazanovskiy 1278 (IRK)	—	KT580712	—	—	—	KT580765
D. spadiceum XVIII	Russia,	Ignatov, Ignatova & Kharzinov s.n.	—	KT580718	—	—	—	KT580771

	Kabardino-Balkariya	(MHA)						
D. spadiceum XIX	Russia, Buryatia	Krivobokov Op.07-Bar09 (UUH)	—	KT580717	—	—	—	KT580770
D. spadiceum XX	Russia, Murmansk Province	Ignatova s.n. (MW)	—	KT580716	—	—	—	KT580769
D. spadiceum XXI	Austria, Schneeberg	Ignatov & Schanzer 05-5054(MHA)	—	KT580727	—	—	—	KT580781
D. spadiceum XXII	Russia, Buryatia	Anenkhonov Op.Ku-02/28 (UUH)	—	KT580715	—	—	—	KT580768
D. spadiceum XXIII	Russia, Buryatia	Anenkhonov Op.Ku-02/33 (UUH)	—	KT580719	—	—	—	KT580772
D. spadiceum XXIV	Russia, Perm Territory	Bezgodov 364 (PPU)	—	KT580714	—	—	—	KT580767
D. spadiceum XXV	Russia, Yakutia (Sakha)	Ignatov & Ignatova 11-3363 (MHA,MW)	—	KT580720	—	—	—	KT580773
D. spadiceum XXVI	USA, Wyoming	Kosovich-Anderson 2394 (MHA ex herb. Kosovich-Anderson)	—	KT580723	—	—	—	KT580777
D. spadiceum XXVII	Russia, Karachaevo-Cherkeyssya	Onipchenko 21/00 (MW)	—	KT580724	—	—	—	KT580778
D. spadiceum XXVIII	Austria	Köckinger 14-989 (Herb. Köckinger)	—	KT580725	—	—	—	KT580779
D. spadiceum XXIX	USA, Wyoming	Kosovich-Anderson 38-04 (MHA ex US)	—	KT580728	—	—	—	KT580782
D. spadiceum XXX	USA, Wyoming	Kosovich-Anderson 2344 (MHA ex herb. Kosovich-Anderson)	—	KT580729	—	—	—	KT580783
D. spadiceum XXXI	USA, Wyoming	Kosovich-Anderson 2356 (MHA ex herb. Kosovich-Anderson)	—	—	—	—	—	KT580784
D. schljakovii I	Russia, Zabaikalskyi Territory	Afonina 9312 (LE)	—	KT580709	—	—	—	KT580762
D. schljakovii II	Russia, Zabaikalskyi Territory	Afonina 1106 (LE)	—	KT580710	—	—	—	KT580763
D. schljakovii III	Russia, Zabaikalskyi Territory	Dudareva s.n. (IRK)	—	KT580711	—	—	—	KT580764
D. schljakovii IV	Russia, Ural	Ignatov s.n. (MHA)	—	KT580705	—	—	—	KT580758

D. schljakovii V	Russia, Zabaikalskyi Territory	Afonina 7212 (LE)	—	KT580701	—	—	—	KT580754
D. schljakovii VI	Russia, Perm Territory	Bezgodov 341 (PPU)	—	KT580702	—	—	—	KT580755
D. schljakovii VII	Russia, Primorskiy Territory	Ignatov 07-280 (MHA)	—	KT580704	—	—	—	KT580757
D. schljakovii VIII	Russia, Buryatia,	Anenkhonov Op.Ku-42/03 (UUH)	—	KT580708	—	—	—	KT580761
D. schljakovii IX	Russia, Khabarovsk Territory	Borisov 92-1 (MW)	—	KT580706	—	—	—	KT580759
D. schljakovii X	Russia, Bashkortostan	Solomesh 18 (MW)	—	KT580703	—	—	—	KT580756
D. schljakovii XI	Russia, Sakhalin	Ignatov & Teleganova 06-760 (MW)	—	KT580721	—	—	—	KT580775
D. schljakovii XII	Russia, Commander Is.	Fedosov 10-3-165 (MW)	—	KT580722	—	—	—	KT580776
D. spurium I	Germany, Bayern	Stech B950821.3 (L)	KM502615	KM502777	—	KM502738	KM502712	KM502690
D. spurium II	Netherlands, Gelderland	Bijlsma 13057 (L)	KM502616	KM502778	KM502653	KM502739	—	KM502691
D. spurium III	Netherlands, Drenthe	Aptroot 69776 (L)	KM502617	KM502779	KM502654	KM502740	—	KM502692
D. tauricum I	Germany, Nordrhein–Westfalen	Stech B911228.3 (L)	KJ651034	KJ651088	KJ650823	KJ650994	KJ650944	KJ650887
D. tauricum II	Netherlands, North Brabant	Smulders 10140 (L)	KJ651043	KJ651099	KJ650841	KJ650995	—	KJ650899
D. tauricum III	Netherlands, Utrecht	Pellicaan L0873254 (L)	KJ651044	KJ651100	KJ650842	KJ650996	—	KJ650900
D. tauricum IV	Netherlands, North Brabant	Buter 73747 (L)	KJ651045	KJ651101	KJ650843	KJ650997	—	KJ650901
D. undulatum I	Finland, North Karelia Prov.	Stech B970824.2 (L)	KJ796626	KJ796604	KJ796537	—	KJ796577	KJ796551
D. undulatum II	Finland, Åland	Hedenäs & Bisang B198289 (S)	KJ796623	KJ796601	KJ796534	KJ796639	KJ796574	KJ796548
D. undulatum III	Sweden, Ångermanland	Hedenäs, Odelvik & Rönblom B199960 (S)	KJ796624	KJ796602	KJ796535	KJ796640	KJ796575	KJ796549
D. undulatum IV	Sweden, Ångermanland	Hedenäs, Odelvik & Rönblom B199806 (S)	KJ796625	KJ796603	KJ796536	KJ796641	KJ796576	KJ796550

D. viride I	Switzerland, Wallis	Greven s.n. (L)	KM502618	KM502780	KM502655	KM502741	KM502713	KM502693
D. viride II	Finland, Karelia Prov.	Stech B970824.3 (L)	KM502619	KM502781	KM502656	KM502742	KM502714	KM502694
D. viride III	Sweden, Småland	Hagström B172407 (S)	KM502620	KM502782	KM502657	KM502743	KM502715	KM502695
D. viride IV	Sweden, Gotland	Lönnell B48734 (S)	KM502621	KM502783	KM502658	—	KM502716	KM502696
Fissidens nobilis	Republic of Korea	W. Kwon, IB-50005 (IN)	NC044155	NC044155	NC044155	NC044155	NC044155	NC044155
Holomitrium arboreum I	Brasil, Parana, Municipio Campina grande do Sul	Stech PA17a (L)	KF423894	KF423994	KF423532	KF423818	KF423740	KF423640
Holomitrium. arboreum II	Brasil, Parana, Municipio Campina grande do Su	Stech PA17b (L)	KF423893	KF423993	KF423531	—	KF423739	KF423639
Holomitrium arboreum III	Brasil, Parana, Municipio Campina grande do Su	Stech PA18 (L)	KF423895	KF423995	KF423533	—	KF423741	KF423641
Holomitrium crispulum	Brasil, Parana, Municipio Campina grande do Su	Stech PA29 (L)	KF423892	KF423992	KF423530	—	KF423738	KF423638
